# Phosphatidylserine externalization, “necroptotic bodies” release, and phagocytosis during necroptosis

**DOI:** 10.1371/journal.pbio.2002711

**Published:** 2017-06-26

**Authors:** Sefi Zargarian, Inbar Shlomovitz, Ziv Erlich, Aria Hourizadeh, Yifat Ofir-Birin, Ben A. Croker, Neta Regev-Rudzki, Liat Edry-Botzer, Motti Gerlic

**Affiliations:** 1 Department of Clinical Microbiology and Immunology, Sackler Faculty of Medicine, Tel Aviv University, Tel Aviv, Israel; 2 Department of Biomolecular Sciences, Weizmann Institute of Science, Rehovot, Israel; 3 Division of Hematology/Oncology, Boston Children’s Hospital, Boston, Massachusetts, United States of America; 4 Department of Pediatrics, Harvard Medical School, Boston, Massachusetts, United States of America; University College London Cancer Institute, United Kingdom of Great Britain and Northern Ireland

## Abstract

Necroptosis is a regulated, nonapoptotic form of cell death initiated by receptor-interacting protein kinase-3 (RIPK3) and mixed lineage kinase domain-like (MLKL) proteins. It is considered to be a form of regulated necrosis, and, by lacking the “find me” and “eat me” signals that are a feature of apoptosis, necroptosis is considered to be inflammatory. One such “eat me” signal observed during apoptosis is the exposure of phosphatidylserine (PS) on the outer plasma membrane. Here, we demonstrate that necroptotic cells also expose PS after phosphorylated mixed lineage kinase-like (pMLKL) translocation to the membrane. Necroptotic cells that expose PS release extracellular vesicles containing proteins and pMLKL to their surroundings. Furthermore, inhibition of pMLKL after PS exposure can reverse the process of necroptosis and restore cell viability. Finally, externalization of PS by necroptotic cells drives recognition and phagocytosis, and this may limit the inflammatory response to this nonapoptotic form of cell death. The exposure of PS to the outer membrane and to extracellular vesicles is therefore a feature of necroptotic cell death and may serve to provide an immunologically-silent window by generating specific “find me” and “eat me” signals.

## Author summary

Necroptosis, a recently discovered regulated form of cell death, is widely considered to be inflammatory due to the absence of specific “find me” and “eat me” signals prior to lytic death. Here, we demonstrate that necroptotic cells generate “find me” and “eat me” signals by exposure of phosphatidylserine on their outer plasma membrane. This was further associated with the release of extracellular vesicles (“necroptotic bodies”) that contain phosphatidylserine, pMLKL (a key necroptotic marker), as well as other proteins. These signals drive recognition and phagocytosis of necroptotic cells to modulate the immune response. The exposure of phosphatidylserine and release of “necroptotic bodies” indicate that apoptosis and necroptosis share some common biochemical and cellular features and highlight the need for new biomarkers to distinguish apoptotic and necroptotic cell death.

## Introduction

Cell death plays a key role in embryonic development, organ function, tumorigenesis, and the initiation and resolution of immune responses [1]. Since the first description of apoptosis as a regulated form of cell death [2], techniques to identify and discriminate apoptotic events from “passive” necrotic events have been developed [3]. Some techniques are based on well-defined morphological changes during apoptosis such as blebbing of the plasma membrane, chromatin condensation, nuclear fragmentation (karyorrhexis), and the formation of apoptotic bodies [2]. Other techniques rely on changes to mitochondrial membrane permeability during apoptosis [4]; these changes result in the release of specific proteins from the intermembrane mitochondrial space, such as cytochrome c and APAF [5]. Other biochemical features of apoptotic cells include the externalization of phosphatidylserine (PS) on the outer plasma membrane leaflet [6], caspase activation, substrate cleavage (e.g., Bid) [7], and nuclear translocation of a caspase-activated DNase that leads to internucleosomal DNA cleavage [8].

Several new types of regulated cell death can now be discriminated from processes previously recognized as passive necrosis. The existence of nonapoptotic regulated forms of necrotic cell death are supported by genetic, biochemical, and pharmacological studies [9]. Necroptosis, a form of necrosis, was originally described as a receptor-interacting protein kinase-1 (RIPK1)-dependent and caspase-independent form of cell death [10]. Recently, necroptosis has been shown to proceed independently of RIPK1 in some settings [11–14]. In vivo, RIPK1 acts as a crucial negative regulator of necroptosis [15–17]. Thus, necroptosis is currently best defined as being a caspase-independent form of cell death that requires the pseudokinase, mixed lineage kinase-like protein (MLKL) [18].

Similar to apoptosis, necroptosis can be induced by several factors including death receptors, toll-like receptors (TLRs), and the intracellular receptors DAI and ZBP1. Unlike apoptosis, necroptosis activation pathways share a common feature: the inhibition of caspase-8 function, which can be achieved by genetic or pharmacological means and is also targeted by viral inhibitors [18].

Apoptosis is widely considered to be immunologically-silent, in contrast to necrotic events, which, by definition, are passive events and lack the regulated release of “find me” and “eat me” signals that are a feature of apoptotic cells. One such signal is the exposure of PS to the outer plasma membrane. As a regulated necrotic event, necroptosis was assumed to lack “eat me” signals, resulting in the constitutive release of cell contents, known as danger-associated molecular patterns (DAMPs), that promote inflammation during infection or chronic inflammatory disease [1,18–20].

In this study, we demonstrate that necroptotic cells expose PS on their outer plasma membrane prior to membrane permeabilization. These findings suggest that PS externalization, and its detection by annexin V (A5), cannot be used as a distinguishing marker between apoptosis and regulated necrosis [21–24].

## Results

### Necroptotic cells expose PS and stain with A5

To test the hypothesis that necroptotic cells lack “eat me” signals such as PS on the outer plasma membrane, we analyzed PS exposure and membrane permeability. Four model systems were used to study these events during apoptosis and necroptosis: (i) a L929 mouse fibrosarcoma cell line, (ii) a U937 human myelomonocytic cell line, (iii) a HaCaT human keratinocyte cell line, and (iv) bone marrow-derived macrophages (BMDMs).

**Fig 1:**
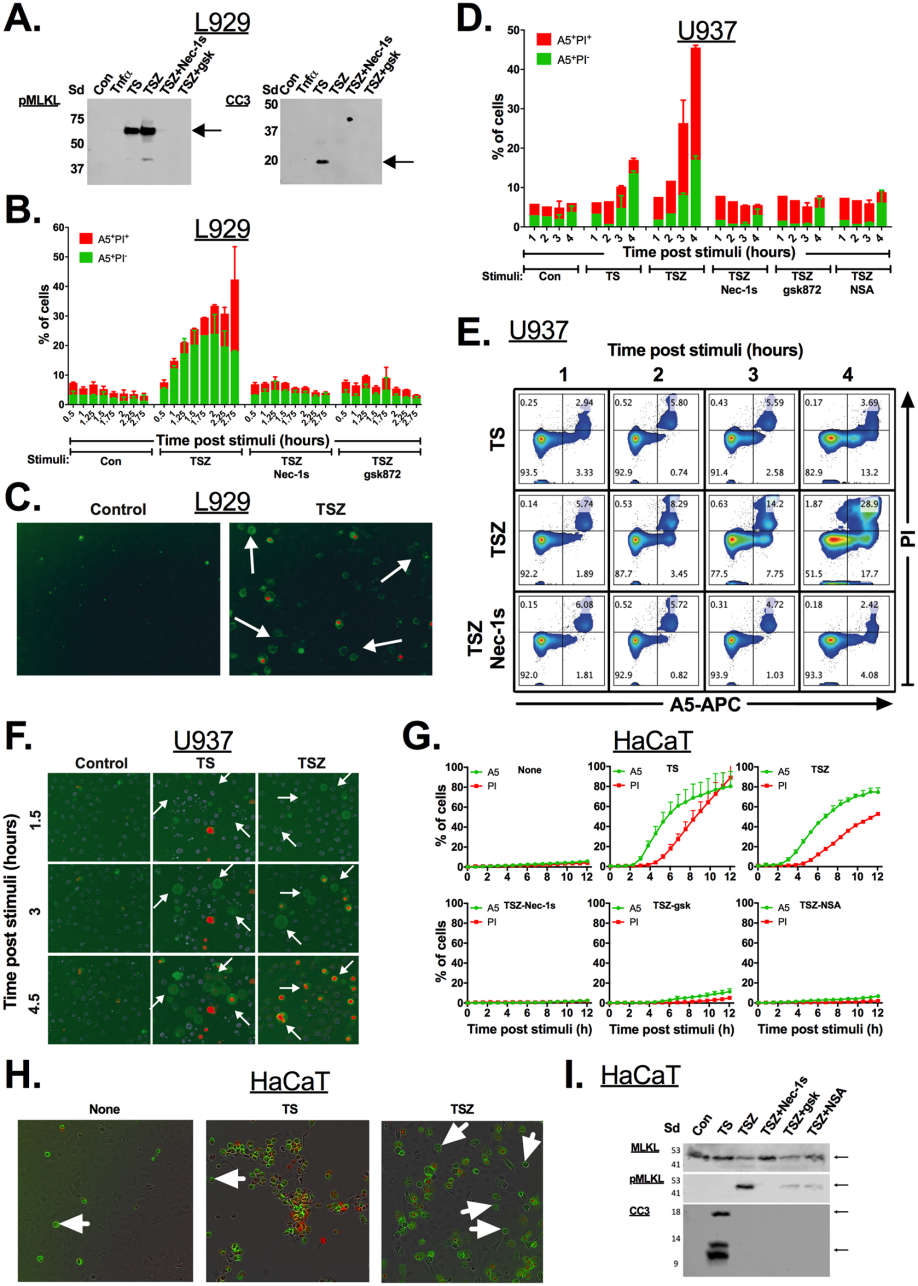
Detection of A5 single-positive cells during necroptosis. **(A–C)** L929 cells were stimulated for either (i) apoptosis (TS), necroptosis (TSZ), or (ii) left untreated (Con). Where indicated, RIPK1 (Nec-1s) and RIPK3 (gsk872) inhibitors were added to the cells 30 minutes prior to TSZ stimulation. **(A)** 10^6^ cells were harvested 3 hours after stimulation of cell death, and cell death key factors pMLKL and CC3 were detected using western blot. **(B)** Cell viability was measured at different time points after stimulation of cell death using A5/PI staining and then analyzed by flow cytometry (mean ± SD). **(C)** Example single A5-FITC-positive necroptotic cells, imaged using live microscopy. **(D–F)** U937 cells were stimulated for either (i) apoptosis (TS), necroptosis (TSZ) or (ii) left untreated (Con). Where indicated, RIPK1 (Nec-1s), RIPK3 (gsk872) and pMLKL (NSA) inhibitors were added to the cells 30 minutes prior to TSZ stimulation. **(D)** Cell viability was measured at different time points after stimulation of cell death using A5/PI staining and then analyzed by flow cytometry (mean ± SD). **(E)** Example smoothed flow cytometry density plots. **(F)** Example single A5-FITC-positive necroptotic cells, imaged using live microscopy. **(G–H)** 3 x 10^4^ HaCaT cells per well in 96-well plate were stimulated, and PI and A5-FITC were added. The plate was placed on IncuCyteZOOM apparatus and 2 images per well were recorded every 30–45 minutes. **(G)** Normalized PI- or A5-positive objects per image. **(H)** Example single A5-positive necroptotic cells, imaged using IncuCyteZOOM apparatus. **(I)** HaCaT cells were harvested, and the cell death key factors pMLKL and CC3 were detected using western blot. Representative data are shown from 1 of at least 2 independent experiments. All raw data for the data summarized under this Fig can be found in S1 Data. A5, annexin V, CC3, cleaved caspase 3; Con, control; FITC, fluorescein isothiocyanate; MLKL, mixed lineage kinase domain-like; Nec-1s, necroptotic inhibitor of RIPK1; NSA, necrosulfonamide; PI, propidium iodide; pMLKL, phosphorylated mixed lineage kinase-like; RIPK1, receptor-interacting protein kinase-1; RIPK3, receptor-interacting protein kinase-3; Sd, protein ladder; SD, standard deviation; TS, TNFα + SMAC mimetic; TSZ, TNFα + SMAC mimetic + zVAD.

Unexpectedly, the induction of necroptosis in L929 cells using TNFα and the SMAC (second mitochondrial-derived activator of caspase) mimetic AZD 5582 dihydrochloride (a pan inhibitor of apoptosis [IAP] inhibitor) (TS), which were reported to undergo spontaneous necroptosis without the need for caspase inhibition [7], generated a mixed population containing phosphorylated mixed lineage kinase-like (pMLKL), cleaved caspase-3 (CC3) and A5-positive propidium iodide (PI)-negative cells (Fig 1A and 1B, S1A and S1B Fig and S1 Data). Since these data in L929 cells may be a result of a mixed phenotypic cell death (e.g., some cells undergo apoptosis and some necroptosis), we blocked caspases in L929 cells. As predicted, caspase inhibition using Z-VAD-FMK (zVAD) in L929 cells prior to treatment with TNFα and SMAC mimetic (TNFα + SMAC mimetic + zVAD [TSZ]) results in a necroptotic phenotype; e.g., pMLKL detection without caspase-3 cleavage (Fig 1A). Nevertheless, the appearance of an A5-positive PI-negative population was not eliminated by caspase inhibition (Fig 1B and 1C and S1A–S1C Fig), suggesting that this method should not be used to distinguish apoptosis from necroptosis. To confirm that cell death in L929 cells was indeed necroptosis, we added the necroptotic inhibitors, Nec-1s (necroptotic inhibitor of RIPK1) and gsk872 (receptor-interacting protein kinase-3 [RIPK3] inhibitor), prior to cell death induction. RIPK1 and RIPK3 inhibitors successfully reduced necroptotic cell death and the detection of pMLKL, as shown in Fig 1A and 1B and S1A–S1C Fig. The necroptotic inhibitors also prevented the detection of an A5-positive population in L929 cells (Fig 1B and S1A–S1C Fig), suggesting that PS externalization occurs downstream of RIPK1 and RIPK3.

To confirm that the A5-positive population in L929 cells is due to activation of a necroptotic pathway, we repeated these experiments using the U937 cell line, which is known to be sensitive to both apoptotic and necroptotic cell death stimuli. Apoptosis in U937 cells using TS for 3 or 6 hours results in caspase-3 cleavage and the appearance of a PI-positive population, without phosphorylation of the key necroptotic effector protein MLKL (S1D Fig). As expected, pretreatment of U937 cells with the pan-caspase inhibitor zVAD prior to stimulation with TNFα and SMAC mimetic (TSZ) results in a switch from apoptosis to necroptosis. This was confirmed by pMLKL translocation to the plasma membrane in the absence of caspase-3 cleavage (S1D Fig). Fig 1D, 1E and 1F show that necroptotic U937 cells become A5-positive prior to staining with PI. This was further confirmed using HaCaT cells (Fig 1G–1I) and BMDMs (S1E Fig). To study the single cell kinetics of these cell death events, we used live cell imaging to examine PS externalization and membrane permeablization using real-time microscopy. Both the microscopy movie data (S1–S10 Movie) and the data presented in Fig 1C, 1F, 1H, and S1C Fig suggest that necroptotic cells expose PS on their outer plasma membrane prior to loss of membrane integrity (detected by A5 and by PI staining).

### Necroptotic cells expose PS to the outer plasma membrane prior to its permeabilization

**Fig 2:**
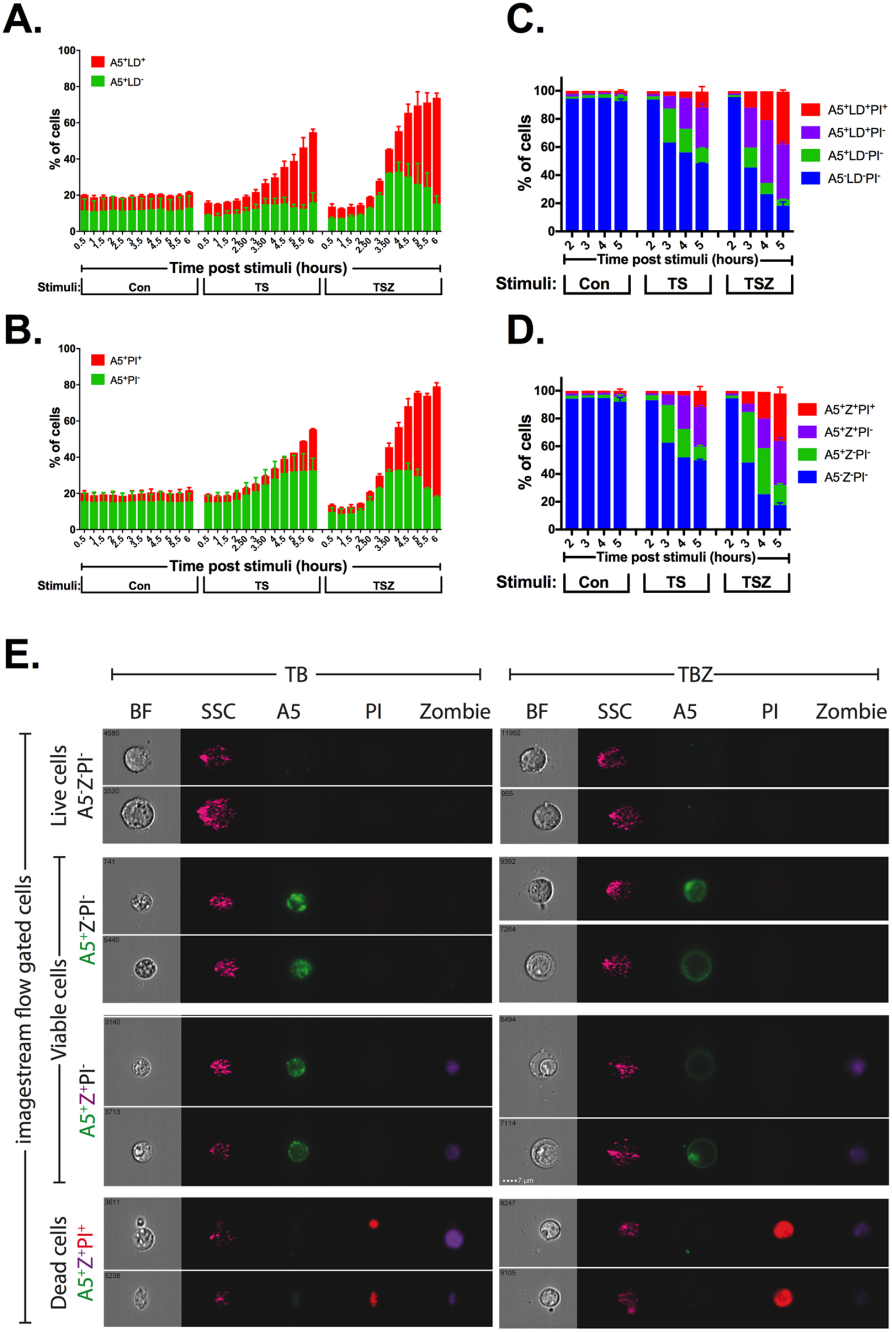
Necroptotic cells expose PS to the outer plasma membrane prior to its permeabilization. U937 cells were stimulated for either (i) apoptosis (TS), necroptosis (TSZ), or (ii) left untreated (Con). **(A–D)** Cell viability was measured at different time points after stimulation of cell death by flow cytometry using **(A)** A5 and LD, **(B)** A5- and PI-staining, **(C)** A5, LD, and PI or **(D)** A5, Z, and PI triple staining. **(E)** Four hours after stimulation of apoptosis (TB) or necroptosis (TBZ), U937 cells were triple stained with A5, Z, and PI, and analyzed by imagestream flow cytometry. Example imagestream flow plots are shown, brightness contrast for each channel between each population was identical. Representative data are shown from 1 of at least 2 independent experiments that were carried out. All raw data for the data summarized under this Fig can be found in S2 Data. A5, annexin V; BF, bright field; Con, control; LD, LiveDead; PI, propidium iodide; PS, phosphatidylserine; SSC, side scatter; TB, TNFα + birinapant; TBZ, TNFα + birinapant + zVAD; TS, TNFα + SMAC mimetic; TSZ, TNFα + SMAC mimetic + zVAD; Z, Zombie.

The cell-impermeant dye, PI, is known to react when binding to either RNA or DNA. Thus, one explanation for our necroptotic-positive A5 staining is that outer membrane integrity is compromised while the organelle and nuclear membranes remain intact. This will result in penetration of A5 into the cytoplasm and binding to PS on the inner plasma membrane while PI staining is still undetected. Thus, to confirm that the A5-positive population is a result of PS exposure to the outer plasma membrane and not due to outer membrane permeabilization prior to organelle and nuclear membrane permeabilization, we first compared a cell-impermeant, amine-reactive dye, LiveDead (LD), prior to PI uptake. As can be seen in Fig 2A and 2B, the combination of A5 staining with LD dye uptake results in similar kinetics to A5/PI staining. Thus, we decided to include the use of a cell-impermeant, amine-reactive dye uptake (LD or Zombie [Z]) into our staining protocol. As can be seen in Fig 2C and 2D, S2 Fig and S2 Data, a population of A5-positive, amine-reactive dye uptake-negative and PI-negative was detected during necroptosis. This new staining protocol further suggested that our necroptotic A5-positive population was a result of PS exposure to the outer plasma membrane and not due to outer membrane permeabilization. The use of 2 different cell-impermeant, amine-reactive dyes, together with A5 and PI, revealed a 2-step mechanism for membrane permeabilization during necroptosis. Firstly, cells lose their outer membrane integrity without losing the integrity of the nuclear membrane, as detected by A5 and cell-impermeant, amine-reactive dye double-positive, PI-negative cells. Secondly, necroptotic cells became triple-positive for A5, cell-impermeant, amine-reactive dye and PI, indicating that nuclear membrane integrity was lost. This was further confirmed using imaging flow cytometry, where the nucleus was still PI-negative in A5-positive Z-positive cells (A5^+^Z^+^PI^-^) prior to becoming triple-positive (A5^+^Z^+^PI^+^) (Fig 2E). Note that the A5 signal was lower in the triple-positive (A5^+^Z^+^PI^+^) apoptotic and necroptotic cells.

**Fig 3:**
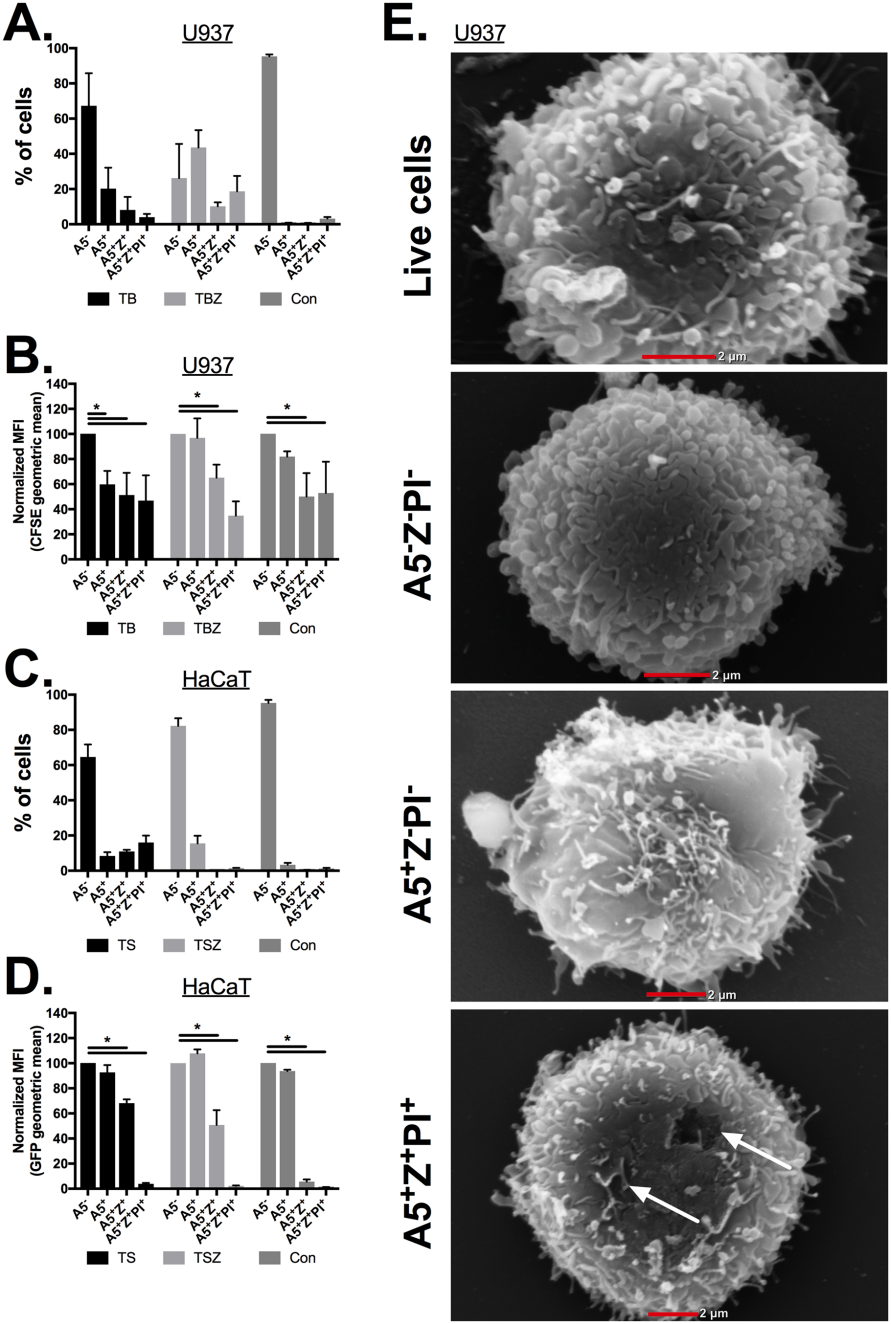
PS-exposed necroptotic cells do not show membrane leakage. **(A–B)** CFSE labeled U937 cells were stimulated for either (i) apoptosis (TB), necroptosis (TBZ) or (ii) left untreated (Con). **(A)** Three hours after stimulation, cell viability was measured using A5, Z, and PI triple staining, and then analyzed by flow cytometry. **(B)** Geometric mean of CFSE fluorescent in the different populations from (A). Representative data are shown from 1 of at least 3 independent experiments that were carried out. **(C-D)** GFP expressing HaCaT cells were stimulated for either (i) apoptosis (TS), necroptosis (TSZ), or (ii) left untreated (Con). **(C)** Nine hours after stimulation, cell viability was measured using A5, Z, and PI triple staining, and then analyzed by flow cytometry. **(D)** Geometric mean of GFP fluorescent in the different populations from (A) were calculated using FloJo software. Representative data are shown from 1 of at least 2 independent experiments that were carried out. **(E)** Necroptosis (TBZ) U937 cells were isolated into 3 different populations according to their A5, Z, and PI triple staining by FACSAria using FACSDiva software (BD Biosciences). Sorted cells and untreated cells (live cells) were fixed and prepared for SEM analysis. Statistic comparisons between each population to the A5^−^ population were carried out for each treatment using ANOVA, followed by a Tukey’s multiple comparison test, * *p* < *0*.*05*. All raw data for the data summarized under this Fig can be found in S3 Data. A5, annexin V; Con, control; CFSE, carboxyfluorescein succinimidyl ester; GFP, green fluorescent protein; MFI, geometric mean fluorescence intensity; PI, propidium iodide; PS, phosphatidylserine; SEM, scanning electron microscopy; TB, TNFα + birinapant; TBZ, TNFα + birinapant + zVAD; TS, TNFα + SMAC mimetic; TSZ, TNFα + SMAC mimetic + zVAD; Z, Zombie.

Another possibility is that the A5-allophycocyanin (APC) molecule (~180 kDa) is smaller than the LD or Z dye reagents (as no information regarding the dimensions of the dye molecules is given by the manufacturers). This may result in faster penetration of A5-APC into the cell via a permeable membrane. Thus, we tested the leakage of proteins during necroptosis using the small protein dye (3kDa) carboxyfluorescein succinimidyl ester (CFSE), or by stably expressing green fluorescent protein (GFP) (a ~30 kDa protein) in cells. In agreement with our hypothesis, CFSE and GFP fluorescence in U937 and HaCaT cells, respectively, did not decrease in the single A5-positive necroptotic cells (TBZ; TNFα + birinapant + zVAD) compared to the A5^-^Z^-^PI^-^ population, while a robust decrease was correlated to both Z and PI uptake (Fig 3A–3D and S3 Data). Analyzing necroptotic cells under scanning electron microscopy (SEM) further confirmed that A5-positive cells retain an intact outer membrane compared to the Z or PI uptake-positive cells (Fig 3E and S3 Fig).

### Necroptotic cells release PS-exposed extracellular vesicles

Apoptotic cells are known to alert neighboring cells (“find me” signals) by the release of apoptotic bodies. These apoptotic bodies are between 0.5–2 μm in size [25], and they consist of cytoplasmic proteins, organelles, and nuclear fractions [26]. These bodies, similar to apoptotic cells, express PS on their outer membrane, which serve as “eat me” signals for phagocytic cells [27].

**Fig 4:**
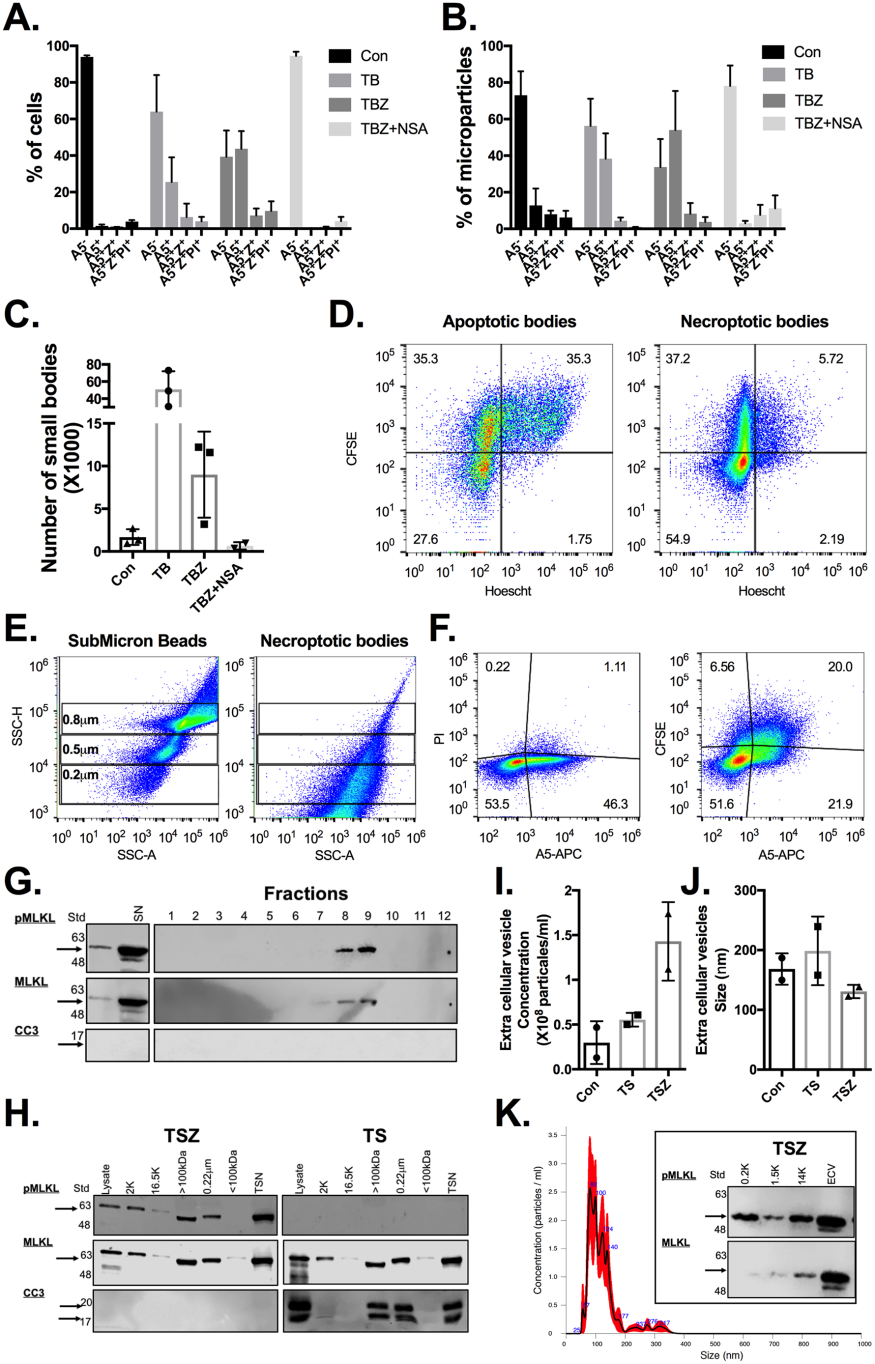
Necroptotic cells release PS-exposed ECVs and pMLKL. **(A-C)** CFSE labeled U937 cells were stimulated for either (i) apoptosis (TB), necroptosis (TBZ), or (ii) left untreated (Con). Three hours after stimulation, cells **(A)** and microparticles **(B)** were divided by their A5, Z, and PI staining, and the geometric mean of CFSE fluorescence in the different populations was then calculated using FloJo software. **(C)** Calculated total number of microparticles in the different treatments. Data are taken from 3 independent experiments. **(D)** CFSE and Hoescht prelabeled U937 cells were stimulated as above. Three hours after stimulation, apoptotic/necroptotic bodies were isolated and then analyzed for CFSE and Hoescht staining using flow cytometry. **(E–F)** ECVs from CFSE labeled U937 necroptotic cells were isolated using a size exclusion column (qEV, ZION), (fractions 7–9). **(E)** ECV (right panel) size was compered to submicron beads of known size (left panel) and **(F)** further stained for A5 and PI, and then analyzed using flow cytometry. **(G)** The different isolated fractions (qEV, ZION) from U937 necroptotic cells were concentrated, and the cell death key factors pMLKL and CC3 were detected using western blot. **(H)** U937 cells were stimulated for apoptosis (TS) or necroptosis (TSZ). Treated cells and supernatants were fractionated as illustrated in S5C Fig. Cell death key factors pMLKL and CC3 were detected in the different fractions using western blot. **(I–K)** 5 x 10^6^ U937 cells were stimulated for either (i) apoptosis (TB), necroptosis (TBZ), or (ii) left untreated (Con). ECVs from treated supernatants were isolated using ultracentrifuge and their concentration **(I)** and size **(J)** were analyzed using NanoSight. **(K)** Example NanoSight histogram and detection of pMLKL in ECVs. All raw data for the data summarized under this Fig can be found in S4 Data. APC, allophycocyanin; A5, annexin V; CC3, cleaved caspase 3; Con, control; CFSE, carboxyfluorescein succinimidyl ester; ECV, extracellular vesicle; MFI, geometric mean fluorescence intensity; MLKL; mixed lineage kinase domain-like; NSA, necrosulfonamide; PI, propidium iodide; pMLKL, phosphorylated mixed lineage kinase-like; PS, phosphatidylserine; Std, protein ladder; SN, supernatant; SSC, side scatter; TB, TNFα + birinapant; TBZ, TNFα + birinapant + zVAD; TS, TNFα + SMAC mimetic; TSN, total supernatant; TSZ, TNFα + SMAC mimetic + zVAD; Z, Zombie; zVAD, Z-VAD-FMK.

To test if a similar mechanism may occur upon necroptosis, we decided to look for “necroptotic bodies.” First, we examined populations of forward scatter (FSC)/side scatter (SSC)-low events by flow cytometry to identify possible “apoptotic bodies” and “necroptotic bodies.” In both apoptotic and necroptotic samples, the amount of FSC/SSC-low populations were significantly increased compared to controls (in the early stages of cell death) and had a comparable A5/Z/PI staining to the reflective normal FSC/SSC cell population (Fig 4A–4C and S4 Data), suggesting that they were apoptotic/necroptotic bodies. We further purified these apoptotic/necroptotic bodies by density gradient centrifugation of supernatants from apoptotic and necroptotic CFSE and Hoescht prelabeled cells. Indeed, this new population contained a large number of “apoptotic bodies,” as detected by their protein content (CFSE^+^) and DNA content (Hoescht^+^), while the necroptosis sample, which was smaller in FSC/SSC, contained proteins (CFSE^+^) without significant DNA content (Hoescht^-^) (Fig 4D). To further study this phenomenon, we collected supernatant from CFSE-labeled necroptotic cells, isolated extracellular vesicles (ECVs) using a size exclusion column (qEV, ZION) and then analyzed them using Attune NxT flow cytometry. First, we confirmed that necroptotic cells release ECVs by comparing the necroptotic vesicle size to fluorescent beads (Fig 4E and S4A Fig). This suggested that the majority of the necroptotic ECV were between 0.2–0.8 μm in size, which is smaller than apoptotic bodies. These “necroptotic bodies” shared other characteristics with apoptotic bodies as they were A5-positive, PI-negative and contained proteins (as detected by CFSE) (Fig 4F and S4B and S4C Fig). Finally, we confirmed that these were ECVs using transmission electron microscopy (TEM) (S5A Fig).

### pMLKL is detected in necroptotic bodies and in supernatant of necroptotic cells

As we found that necroptotic bodies detected from cultures of necroptotic cells contained proteins, we examined their protein content by investigating key markers of necroptosis. pMLKL was detected in the necroptotic supernatant (Fig 4G) and in ECV fractions 7–9 (as a control, we detected caspase-3 cleavage in apoptotic supernatant and in their ECV fractions 7–9, S5B Fig). This suggests that pMLKL can be released to the extracellular environment as part of necroptotic bodies. To further fractionate cellular and subcellular particles, we applied additional centrifuge steps to cells, cell debris, and ECVs (S5C Fig). pMLKL was detected in all supernatant fractions, but was lost by filtering molecules under 100 kDa, suggesting that pMLKL is either in necroptotic bodies or as part of a complex (Fig 4H). Thus, we continued and isolated ECVs using ultracentrifugation (Fig 4I–4K) or another commercial kit (ExoQuick) (S5D–S5F Fig) and analyzed them using NanoSight. Necroptotic cells (TSZ) released a similar size and a higher number of ECVs than untreated cells (Fig 4I and 4J and S5D and S5E Fig). Furthermore, these ECVs also contained pMLKL, as detected by immunoblot (Fig 4K and S5F Fig).

### PS-exposure during necroptosis is dependent on pMLKL translocation to membrane

**Fig 5:**
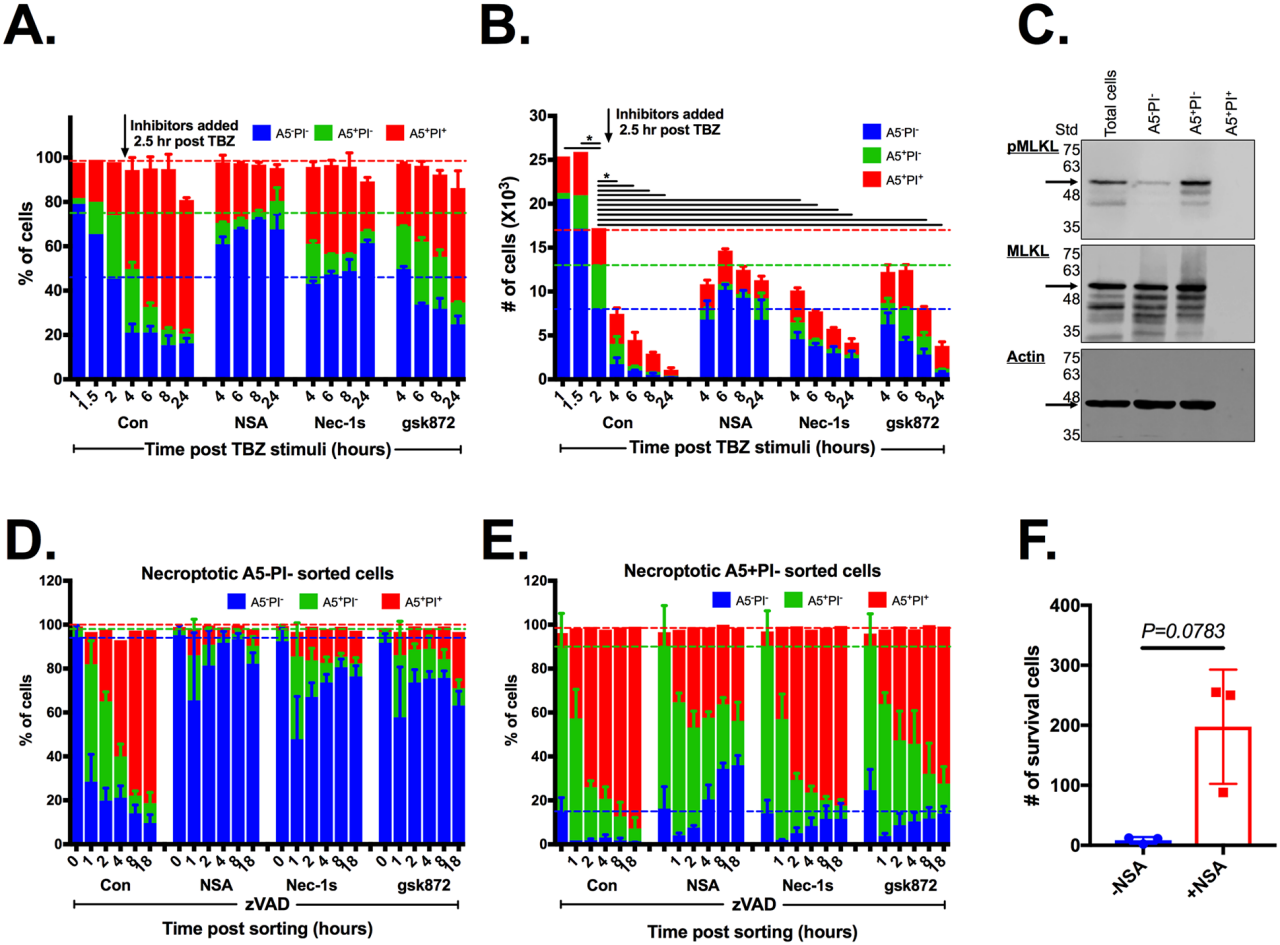
Necroptotic A5 single-positive cell death requires pMLKL membrane translocation. U937 cells were stimulated for necroptosis using TNFα, birinapant (SMAC mimetic) and zVAD (TBZ). After 2 hours, cells were treated with necroptotic inhibitors RIPK1 (Nec-1s), RIPK3 (gsk872), and pMLKL (NSA), or left untreated (Con). **(A)** Cell viability was measured at different time points after stimulation of cell death using A5/PI staining and then analyzed by flow cytometry (mean ± SD). **(B)** Cell counts were measured using the Attune NxT flow cytometer (mean ± SD). Statistic comparisons between total cell numbers in each group to total cell numbers at the 2 hour time point in the control group was carried using ANOVA, followed by a Tukey’s multiple comparison test, * *p* < *0*.*05*. Data are representative of 1 experiment from at least 3 independent ones. **(C–F)** U937 cells were stimulated as in (A) for 2 hours, prior to A5/PI staining. Cells were sorted into live cells (double-negative) or PS-exposed-necroptotic (A5-positive PI-negative). **(C)** Necroptotic cell death key factor pMLKL was detected in the different sorted populations using western blot. Cell viability was measured at different time points after sorting in the A5^−^PI^−^ **(D)** and A5^+^PI^−^ **(E)** populations, using A5/PI staining, and then analyzed by flow cytometry (mean ± SD). Where indicated, RIPK1 (Nec-1s), RIPK3 (gsk872), and pMLKL (NSA) inhibitors were added to the collection tubes. Dashed lines represent the state of cells prior to the addition of inhibitors. Data are taken from 3 independent experiments. **(E)** U937 cells were stimulated and sorted into PS-exposed-necroptotic (A5-positive PI-negative) as above. Viable cells (A5^−^PI^−^) were measured and counted at 6 days after sorting. Data are taken from 2 independent experiments. (mean ± SD). Statistic comparisons between total cell numbers was carried using parametric paired Student *t* test. All raw data for the data summarized under this Fig can be found in S5 Data. A5, annexin V; Con, control; Nec-1s; necroptotic inhibitor of RIPK1; MLKL, mixed lineage kinase domain-like; NSA; necrosulfonamide; PI; propidium iodide; pMLKL; phosphorylated mixed lineage kinase-like protein; PS, phosphatidylserine; RIPK1, receptor-interacting protein kinase-1; RIPK3, receptor-interacting protein kinase-3; SD, standard deviation; SMAC, second mitochondrial-derived activator of caspase; Std, protein ladder; TBZ, TNFα + birinapant + zVAD; zVAD, Z-VAD-FMK.

Necrosulfonamide (NSA), which binds pMLKL and inhibits pMLKL membrane translocation [28] prior to induction of necroptosis, inhibited necroptosis and PS exposure (Fig 1, S1 Fig and S1 Data). To further understand the role of pMLKL translocation to the plasma membrane during PS exposure, we repeated our experiments with the addition of necroptotic inhibitors at different time points after stimulation. Addition of Nec-1s, up to 2 hours after necroptosis induction, partially prevented the death of A5/PI double-negative cells (Fig 5A and 5B, S6 Fig and S5 Data). In comparison, NSA prevented the death of A5/PI double-negative cells for a longer timeframe after necroptosis induction (S6 Fig). When NSA was added after necroptosis induction, A5/PI double-positive cell numbers were unchanged, while A5 single-positive cells reduced in number and live cells (A5/PI double-negative) were increased in number (Fig 5A and 5B). This alteration in cellular composition suggests that A5-positive PI-negative necroptotic cells are not committed to die and that pMLKL translocation to the outer membrane is not only required for the appearance of PS on the external membrane but it is also required for subsequent loss of viability. To confirm these data, we fractionated necroptotic cells according to their A5 and PI staining and determined the levels of pMLKL. pMLKL detection in total necroptotic cells (first lane) can be attributed primarily to the single A5-positive population (third lane) and not to the A5/PI double-negative (second lane) or the A5/PI double-positive (fourth lane) populations (Fig 5C). Then, we isolated PS-exposed necroptotic cells prior to the addition of necroptotic inhibitors. NSA but not Nec-1s or gsk872 partially rescued PS-exposed necroptotic cells (e.g., single A5-sorted necroptotic cells) for a short time (Fig 5D and 5E). Furthermore, the single A5-sorted necroptotic cells, which were treated with NSA for the first 24 hours post sorting, showed a long-term survival advantage (Fig 5F).

### Phagocytosis of PS-exposed necroptotic cells

**Fig 6:**
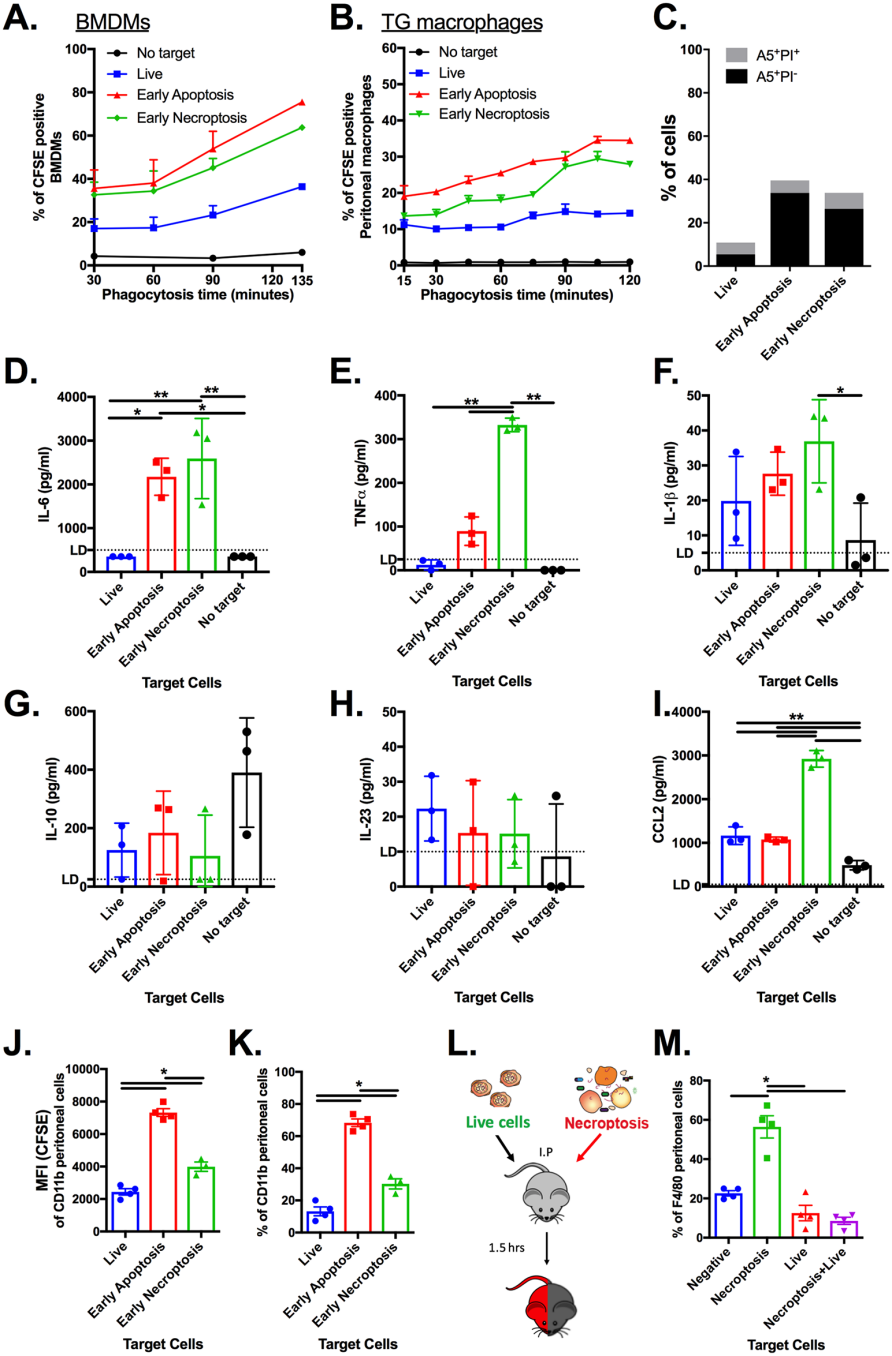
Phagocytosis of PS-exposed-necroptotic cells. **(A)** U937 cells were first stained with CFSE prior to stimulation for apoptosis and necroptosis using a combination of TNFα, birinapant (SMAC mimetic), and zVAD. PS exposure was tested every 30 minutes until exposure reached 40% in both the apoptotic and necroptotic samples (determined by A5/PI staining). Cells were washed twice and resuspended in DMEM before adding on IFN-γ treated BMDMs at a 2.5:1 ratio. Phagocytosis was analyzed by flow cytometry (mean ± SD). BMDMs with or without addition of live cells served as negative controls. Data are taken from 3 independent experiments. **(B)** U937 cells treated as above were added on TG peritoneal macrophages at a 2.5:1 ratio, and phagocytosis was analyzed by flow cytometry (mean ± SD). **(C)** Viability of the U937 cells, which were used as target to (B), is shown. TG peritoneal macrophages with no addition of cells or with addition of live cells served as negative controls. **(D–I)** Supernatants from the phagocytic TG peritoneal macrophages from (B) were collected and then analyzed for cytokines and chemokines using ELISAs. Statistic comparisons between each injected target cells were carried using ANOVA, followed by a Tukey’s multiple comparison test, * *p < 0*.*05*. **(J–K)** U937 cells were left untreated or stimulated for apoptosis or necroptosis. PS exposure was tested every 30 minutes until reaching 40% in apoptosis and necroptotic samples (determined by A5/PI staining). 2 x 10^6^ cells in 100 μl per mouse were IP injected. One hour after injection, CD11b peritoneal cells were analyzed for CFSE fluorescence **(J)** and phagocytosis of CFSE target cells **(K)** by flow cytometry (*N* = 4, mean ± sem). Statistic comparisons between each injected target cells were carried using ANOVA, followed by a Tukey’s multiple comparison test, * *p < 0*.*05*. **(L)** Illustration of competitive in vivo phagocytosis assay. CFSE- or Hoescht-stained L929 cells were left untreated or stimulated for necroptosis (TSZ). PS exposure was tested every 30 minutes until reaching 40% in necroptotic samples (determined by A5/PI staining). A 1:1 ratio mix of live and necroptotic cells was IP injected (total of 2 x 10^6^ cells in 100 μl per mouse). (M) One hour after injection, F4/80 peritoneal cells were analyzed for phagocytosis of the live and/or necroptotic cells by flow cytometry (*N* = 4, mean ± sem). Statistic comparisons between each injected target cells populations were carried using ANOVA, followed by a Tukey’s multiple comparison test, * *p < 0*.*05*. All raw data for the data summarized under this Fig can be found in S6 Data. A5, annexin V; BMDM, bone marrow-derived macrophages; CFSE, carboxyfluorescein succinimidyl ester; DMEM, dulbecco’s modified eagle’s medium; IP, intraperitoneally; PI, propidium iodide; MFI, mean fluorescence intensity; PS, phosphatidylserine; SD, standard deviation; SMAC, second mitochondrial-derived activator of caspase; TG, thioglycolate; zVAD, Z-VAD-FMK.

To investigate if PS exposure during necroptosis can act as an “eat me” signal, similar to its role in apoptotic cells, we set up 4 phagocytosis models. In the first 2 models, we compared the ability of mouse macrophages (BMDMs and thioglycolate [TG] peritoneal macrophages) to phagocytose PS-exposed necroptotic cells ex vivo. Both BMDMs and TG peritoneal macrophages were able to phagocytose necroptotic cells more efficiently than live cells but less efficiently than apoptotic cells (TG peritoneal macrophages) (Fig 6A–6C, S7A–S7D Fig and S6 Data). Next, to understand the consequences of phagocytosis on inflammation, we measured the secretion of proinflammatory cytokines (IL-6, TNFα, IL-1β, and IL-23), the anti-inflammatory cytokine IL-10, and the CCL2 chemokine from peritoneal macrophages. Phagocytosis of necroptotic cells by TG peritoneal macrophages results in high secretion of the proinflammatory cytokines IL-6 and TNFα in comparison to live targets or no target cells (Fig 6D and 6E and S7E and S7F Fig). When compared to apoptotic cell targets TNFα, but not IL-6, was also significantly elevated by phagocytosis of necroptotic cells. IL-1β was significantly elevated compared to macrophages fed with no target cells (Fig 6F and S7G Fig), while IL-23 and the anti-inflammatory cytokine IL-10 were both unchanged in all samples (Fig 6G and 6H and S7H and S7I Fig). Similar to TNFα, levels of the chemokine CCL2 (monocyte chemoattractant protein 1 [MCP1]), which attract monocytes and macrophages to clear dying cells, was significantly elevated compared to macrophages fed live cells or apoptotic cells (Fig 6I and S7J Fig).

Finally, we measured phagocytosis in vivo by injecting PS-exposed necroptotic cells into the peritoneal cavity of mice. In agreement with our ex vivo experiments, and as seen in Fig 6J and 6K, PS-exposed necroptotic cells were more efficiently phagocytosed than live cells but less efficiently phagocytosed than apoptotic cells. To further eliminate any extrinsic effects possible using the in vivo phagocytosis assay, we competed live and PS-exposed necroptotic cells at a 1:1 ratio (Fig 6L). As summarize in Fig 6M, live cells were outcompeted by PS-exposed necroptotic cells regardless of the labeling dye that was used (S7K–S7M Fig). Taken together, these experiments confirm that PS exposure during necroptosis may act as an “eat me” signal to alter the immune system.

## Discussion

Phagocytosis of apoptotic cells is an established negative regulator of inflammation during normal development and homeostasis. During apoptosis, which restricts content leakage, cells generate “find me” and “eat me” signals to stimulate rapid phagocytic clearance [29]. One such signal is the exposure of PS to the outer plasma membrane. However, when the number of apoptotic cells overwhelms phagocytosis in tissues, secondary “accidental” necrosis and inflammation can occur [30], leading to an autoimmune response [31,32]. Finally, it is assumed that phagocytes are not recruited in the absence of apoptotic signals, and that DAMPs are released from necroptotic and necrotic cells to induce inflammation.

Here, we demonstrate that there is a short time interval during which necroptotic cells expose PS before plasma membrane integrity is lost. Several previous studies have suggested that PS is exposed during nonapoptotic cell death. For example, Krysko et al. used immunogold labeling to show that PS is exposed to the outer plasma membrane leaflet during oncosis [33]. Ferraro-Peyret et al. showed that PS exposure during apoptosis of primary T-lymphocytes proceeds in a caspase-independent manner [34]. Sawai and Domae showed that death of U937 stimulated with TNFα zVAD and de novo protein translation inhibitor CHX can’t be discriminate from apoptotic cells by A5 staining [35]. All 3 studies used models of cell death that would now be considered to be necroptosis. Krysko et al. used a caspase-8-deficient and bcl-2 overexpressing cell line, while Ferraro-Peyret et al. inhibited caspase activity using zVAD prior to FasL stimuli, which normally engage the intrinsic apoptotic cell death pathway.

Our results indicate that the addition of NSA to A5-positive cells to inhibit pMLKL membrane translocation also rescues cell death. This is not the case for apoptosis because NSA failed to rescue A5-positive PI-negative apoptotic cells. We found that NSA-mediated rescue of purified PS-exposed necroptotic cells was only partial, since some cells died during the first hour after sorting. We speculate that this partial rescue was an issue of synchronicity; cells undergo necroptosis at different times poststimulation and cannot be rescued as membrane damage advances. Indeed, after the initial death observed after sorting (e.g., 2 hours), NSA completely inhibited cell death.

Externalization of PS to the outer surface of the plasma membrane during apoptosis is required for the recognition and engulfment of dying cells [36]. Our results suggest that necroptotic cells might also be recognized and phagocytosed. Similarly, Brouckaert et al. showed, using modified L929 cells, that necrotic cells are also phagocytosed in a PS-dependent manner [37]. Our finding that necroptotic cells may also release PS-exposed ECVs, dubbed “necroptotic bodies,” which contain proteins, including pMLKL, together with our finding of “free” pMLKL outside the cells, raises the possibility of a specific alert system or “find me” signal for necroptotic immune recognition. Future studies will be needed to fully understand the mechanisms and physiological importance of this finding. Furthermore, the finding that pMLKL is rapidly released to the outside of cells may also explain the challenging detection of pMLKL in vivo.

Our results also challenge one of the most widespread methods that is used to distinguish apoptosis from necrosis: the detection of PS exposure by A5 staining and permeabilization of the plasma and nuclear membranes with DNA staining [3]. We demonstrate that this method should be avoided as a means of discriminating apoptosis and regulated nonapoptotic cell death.

MLKL inhibition appeared more capable of reversing the fate of necroptotic cells than did inhibitors of RIPK1 and RIPK3. PS-exposed necroptotic cells that were treated with NSA lost A5 staining over time, indicating that they either internalized PS to the inner leaflet of the plasma membrane (Fig 5B and 5E) or that they release this PS-exposed membrane to the extracellular environments as ECVs (Fig 4). This suggests that targeting pMLKL translocation will be more beneficial for reversing necroptotic events than targeting RIPK1 and RIPK3 activation in necroptotic cells that had already exposed PS to their outer membrane.

Interestingly, during the revision preparation of this manuscript, Gong et al. reported, using a necroptosis dimerization model, that PS is exposed during necroptosis by a budding mechanism which is dependent on the endosomal sorting complexes required for transport (ESCRT) machinery [38]. Thus, in the future, it will be interesting to investigate if the same mechanism is involved in the formation of the “necroptotic bodies” described here.

We found that the externalization of PS by necroptotic cells drives recognition and phagocytosis. This phenomenon provides a window for specific “find me” and “eat me” signals from necroptotic cells to engage phagocytic cells and modulate the inflammatory response. Furthermore, it seems that the necroptotic “find me” and “eat me” signals differ from apoptotic ones as can be seen by the slower kinetics of phagocytosis. This slower kinetics may also allow for higher levels of TNFα and CCL2 secretion by macrophages that feed upon necroptotic cells in comparison to apoptotic cells (Fig 6). In conclusion, our findings that necroptotic cells also expose PS and release “necroptotic bodies” indicates that there are also other common features between apoptosis and necroptosis. Moreover, as our knowledge of regulated necrosis continues to expand, additional assumptions that separate different forms of cell death will need to be reassessed.

## Materials and methods

### Ethics

All animal experiments complied with the regulatory standards of, and were approved by, the institutional animal ethics committee at Tel Aviv University.

### Reagents

Unless stated otherwise, all cell culture reagents were purchased from Gibco (Thermo Fisher Scientific, Waltham, MA, USA). Recombinant human TNFα was purchased from PeproTech (Rocky Hill, NJ, USA). The SMAC mimetics, AZD 5582 dihydrochloride, birinapant, and the MLKL inhibitor, NSA, were purchased from Tocris Bioscience (Bristol, UK). The pan-caspase inhibitor, Z-VAD-FMK (zVAD), the RIPK1 inhibitor, Nec-1s, and the RIP3 inhibitor, gsk872, were purchased from Calbiochem (Merck Millipore, MA, USA). Apoptosis kits (A5-FITC and A5-APC) were purchased from MBL International (Woburn, MA, USA). Cell-impermeant, amine-reactive dye uptake (LD-Violet) was purchased from MolecularProbs (Thermo Fisher Scientific, Waltham, MA, USA). Halt protease and the phosphatase inhibitor cocktail EDTA-free was purchased from Pierce Biotechnology (Thermo Fisher Scientific, Waltham, MA, USA). Commercial antibodies were purchased from Abcam (Cambridge, UK) (anti-human-pMLKL, 187091, and anti-mouse-pMLKL, 196436), Merck Millipore (total MLKL, MABC604), BD (RIPK1, 610458), and from Cell Signaling Technology (Danvers, MA, USA) (cleaved caspase-3, 9961). HRP-conjugated secondary antibodies were purchased from Jackson ImmunoResearch Labs (West Grove, PA, USA).

### Cell culture

U937 cells were grown in RPMI culture medium containing 10% fetal bovine serum (FBS), 1% penicillin-streptomycin and 1% 4-(2-hydroxyethyl)-1-piperazineethanesulfonic acid (HEPES), as described previously [39]. L929 and HaCaT cell TG macrophages were grown in DMEM culture medium containing 10% FBS, 1% penicillin-streptomycin, and 1% HEPES. BMDMs were obtained as previously described [40].

### Generating GFP HaCaT cells

To generate a GFP stably expressing HaCaT cell line, HaCaT cells were infected with lentivirus particles containing pLL3.7-CMV-T2A-EGFP vectors with the addition of polybrene (8 μg/μL). GFP-positive cells were sorted (FACSAria) 3 weeks later.

### Mice

C57BL/6J mice were used for generating BMDMs, TG macrophages, and for the in vivo phagocytosis assay. All animal experiments complied with the regulatory standards of, and were approved by, the institutional animal ethics committee at Tel Aviv University.

### Cell death stimuli

Cell death in U937 cells (5 x 10^5^/ml), L929 cells (2.5 x 10^5^/ml) and BMDMs (5 x 10^5^/ml) was induced by addition of TNFα (20 ng/ml) and SMAC mimetic (20 μM), while necroptosis was induced by the addition of zVAD (10 μg/ml) 30 minutes prior to the addition of TNFα and SMAC mimetic. When used, Nec-1s (5 μM), gsk872 (5 μM), and NSA (1 μM) were added 30 minutes prior to the addition of TNFα and SMAC mimetic.

### Assessment of apoptosis and necroptosis

Cell death was determined by PI uptake, A5 and PI staining, and A5 and cell-impermeant, amine-reactive dye uptake (LD or Z) using an automatic cell counter (Countess II, Thermo Fisher Scientific), real-time fluorescence microscopy (EVOS Fl Auto, Thermo Fisher Scientific), flow cytometry (Attune NxT, Thermo Fisher Scientific), and imagestream flow cytometry (Amnis ImageStream X, EMD LTD). The exact mechanism of cell death (apoptosis vs necroptosis) was further determined by the appearance of CC3 (17kDa and 19kDa) and pMLKL (54kDa) by western blot.

### Live imaging assay by IncuCyteZOOM

HaCaT cells were seeded overnight (3 x 10^4^ HaCaT cells per well in a 96-well plate), then the media was replaced with fresh media containing stimulants, PI, and A5-FITC. The plate was placed on IncuCyteZOOM apparatus, and 2 images per well were recorded every 30–45 minutes. Data was analyzed using IncuCyteZoom2016B analysis software. Images and movies were then directly exported from the analysis software. The number of PI- or A5-positive objects per image was also directly exported to GraphPad Prism software. When PI- or A5-positive cells are given as percentages in the text, normalization was performed by GraphPad Prism according to the maximal A5-positive object count.

### SEM

For SEM analysis, U937 cells were treated to induce necroptosis (TNF, birinapant, and zVAD). The percentages of A5/PI-positive cells were monitored by FACS. Two hours after induction, when the cells were 40% positive for A5, the cells were sorted into 3 populations by FACSAria using FACSDiva software (BD Biosciences): double-negative (A5^−^PI^−^), single-positive (A5^+^PI^−^), and double-positive (A5^+^PI^+^). At least 7 x 10^4^ sorted cells were collected from each population. The cells were washed once with PBS and centrifuged on 13-mm cover glass (Mensel-Glaser, Thermo Fisher Scientific). Untreated U937 cells were used as controls. Samples were fixed in 2.5% glutaraldehyde in PBS for 24 hours at 4°C. After washing with PBS, the samples were postfixed in 2% osmium tetroxide solution for 2 hours at 4°C and dehydrated by successive ethanol treatments. After critical point drying (Balzer's critical point drier), the samples were mounted on aluminum stubs and sputter-coated (SC7620, Quorum) with gold. Images were captured on the SEM (JCM-6000, JEOL).

### TEM

For TEM analysis, U937 cells were treated to induce necroptosis (TNF, birinapant, and zVAD). The percentages of A5/PI-positive cells were monitored by FACS. Four hours after induction, when the cells were 50% positive for PI, the cells were centrifuged and the supernatant was collected. Equal amounts of untreated U937 supernatant were used for controls. ECVs were isolated using a qEV Size Exclusion Column (IZon science). The samples were concentrated by centrifugal filtration with 3 kDa-cutoff Amicon ultra-filtration tubes (EMD Millipore) to a final volume of 50 μl. Samples were adsorbed on formvar/carbon coated grids and stained with 2% aqueous uranyl acetate for 30 s. Samples were examined using a JEM 1400plus TEM (Jeol, Japan).

### Assessment of phagocytosis

For ex vivo experiments, U937 cells were first stained with CFSE prior to apoptosis and necroptosis stimulation using a combination of TNFα, birinapant (SMAC mimetic), and zVAD. PS exposure was tested every 30 minutes until reaching 40% A5 positivity in both apoptotic and necroptotic samples (as determined by A5/PI staining). Cells were washed twice, resuspended in DMEM, and then added to IFN-γ treated BMDMs at a 2.5:1 ratio and analyzed for phagocytosis by flow cytometry at different time points. For in vivo experiments, CFSE or Hoescht stained L929 cells were left untreated or stimulated for necroptosis (TSZ). PS exposure was tested every 30 minutes until reaching 40% A5 positivity in necroptotic samples (determined by A5/PI staining). Live and necroptotic cells were washed twice and resuspended in PBS at 1:1 ratio before IP injection (total of 2 x 10^6^ cells in 100 μl PBS per mouse). One hour after injection, peritoneal cells were collected, stained for F4/80 and CD11b expression, and analyzed for phagocytosis of the live and/or necroptotic cells by flow cytometry.

### Purification of ECVs

Cell growth media was collected from cell cultures, and ECVs were purified by commercial kits (qEV or ExoQuick) or by ultracentrifugation method. For ultracentrifugation, cellular debris were first removed by centrifugation at 1,500 g, 3000 g, and 10,000 g. The supernatant was concentrated using a Vivaflow 100,000 MWCO PES (Sartorious Stedium) and centrifuged at 150,000 g to pellet nanovesicles. OptiPrep density gradient performed as previously described [41].

### Fractionation of cytosolic and membrane proteins

Phase partitioning of cytosolic and membrane proteins was performed as previously described with slight modification [42]. Briefly, cell death lysates were separated into hydrophobic and hydrophilic fractions by using Triton X-114 (TX-114; Sigma Chemical, St Louis, MO, USA). Precondensed TX-114 was added to give a final concentration of 1% wt/vol, and proteins were solubilized for 15 minutes at 4°C. The TX-114-solubilized material was incubated for 5 minutes at 37°C in order to induce rapid condensation of the TX-114. The cloudy suspension was centrifuged at 25°C for 5 minutes at 1,500 g. The resulting upper phase (aqueous) was carefully transferred to a second tube and brought to 1% TX-114 by the addition of condensed TX-114, whereas the lower phase (TX-114) was brought to the original volume with RIPA. Both fractions were then dissolved by incubation for 5 minutes at 4°C; phase partitioning, as described above, was repeated 3 times, to obtain fractions of the desired purities.

TX-114 was removed by adding cold methanol to the final condensed TX-114, and centrifuged at 4°C for 10 minutes at 13,000 g. The pellet was dried out and dissolved in SDS*1.

### Western blot analyses of proteins

Cells were collected and centrifuged for 5 minutes at 400 g (4°C) in order to separate cells from supernatant. Cells were lysed using RIPA buffer in the presence of protease and phosphatase inhibitors at 4°C for 15 minutes. Lysed cells were loaded onto gels, and western blot was performed using the specific antibodies.

### Statistics

Unless otherwise specified, data are presented as mean ± standard deviation (SD). Comparisons were performed using Student *t* tests or ANOVA, followed by a Tukey’s multiple comparison test.

## Supporting information

S1 Fig (relevance to Fig 1). **(A-C)** L929 cells were stimulated using TNFa (T), SMAC mimetic (S) and zVAD (Z) as indicated or left unstimulated (Con). Where indicate RIPK1 (nec1s) and RIPK3 (gsk872) inhibitors were added to the cells 30 minutes prior to TSZ stimulation. **(A)** Cell viability was measured at different time point post cell death stimulation using annexin V/PI staining and analyzed by flow cytometry (mean ± sd). **(B)** Example of the flow cytometry smooth density plots are shown. **(C)** Example of the single A5-FITC-positive necroptotic cells using live microscopy are shown. **(D)** Three and six h post stimulation 10^6^ U937 cells were harvested and two fractions were produced from every sample: Aqua (Aq)–hydrophilic fraction and Detergent (Det)—hydrophobic fraction. The kinetics of cell death key factors pMLKL, MLKL, RIPK1 and cleaved caspase 3 (CC3) were detected using western-blot. Sd–protein ladder. **(E)** BMDMs were stimulated for apoptosis (TB) and necroptosis (TBZ) or left unstimulated (Con). Where indicate RIPK1 (nec1s) and inhibitor was added to the cells 30 minutes prior to TBZ stimulation.
(TIFF)

S2 Fig (relevance to Fig 2). U937 cells were stimulated for either (i) apoptosis (TB), necroptosis (TBZ) or (ii) left untreated (Con). MLKL (NSA) inhibitor was added to the cells 30 minutes prior to TBZ stimulation. Illustration of flow cytometry gating strategy for A5, Zombie and PI (left panels) and A5, LiveDead and PI (right panels) triple staining. First, single cells were analyzed for A5 positivity (top histograms). A5 positive cells (green arrows) were further analyzed for Zombie and PI (lower left smooth density plots) and LiveDead and PI (lower right smooth density plots).
(TIFF)

S3 Fig (relevance to Fig 3). Necroptosis (TBZ) U937 cells were isolated into three different population according to their A5, Zombie and PI triple staining by FACSAria (BD Biosciences). Sorted cells and untreated cells (live cells) were fixed and prepared for SEM analysis.
(TIFF)

S4 Fig (relevance to Fig 4). Extracellular vesicles (ECVs) from supernatants from CFSE labeled U937 necroptotic cells were isolated using size exclusion column (qEV, ZION). **(A)** The different fractions particles size was compared to known submicron beads. **(B-C)** The different fractions particles were further stained for A5 and PI and analyzed for A5, PI and CFSE using flow cytometry.
(TIFF)

S5 Fig (relevance to Fig 4). **(A)** Extra cellular vesicles (ECV) were isolated from necroptotic (TBZ) U937 cells by qEV Size Exclusion Column (IZon science). ECVs were prepared for transmission electron microscope (TEM) and images were captured on the JEM 1400plus transmission electron microscope (Jeol, Japan). **(B)** Supernatants from U937 apoptotic cells was fractionated using size exclusion column (qEV, ZION) and the cell death key factors pMLKL and cleaved caspase 3 (CC3) were detected using western-blot (SN–supernatants, Std–protein ladder). **(C)** Illustration of the fractionation of U937 treated cells and supernatants from Fig 4H. TSN-Total supernatant; SN- supernatant. **(D-F)** 5x10^6^ U937 cells were stimulated for either (i) apoptosis (TB), necroptosis (TBZ) or (ii) left untreated (None). ECVs from treated supernatants were isolated using ExoQuick kit (SBI, USA) and their concentration **(D)** and size **(E)** was analyzed using NanoSight. **(F)** Detection of pMLKL in the ECVs is shown.
(TIFF)

S6 Fig (relevance to Fig 5). **(A)** L929 cells were stimulated for necroptosis (TSZ). From 30 minutes post stimulation, every 15 minutes cell viability was measured using A5/PI staining (indicate by «) prior to addition of RIPK1 (nec1s) or RIPK3 (gsk872) inhibitors. 2.75 hours post necroptosis induction cell viability was measured in all treatment using A5/PI staining and analyzed by flow cytometry. **(B)** U937 cells were stimulated for necroptosis (TSZ). Every hour post necroptosis stimulation cell viability was measured as below (indicate by «) prior to addition of RIPK1 (nec1s) or pMLKL (NSA) inhibitors. Six hours post necroptosis induction cell viability was measured in all treatment using A5/PI staining or A5/LiveDead (indicate as LMI positive) and analyzed by flow cytometry. **(C-D)** U937 cells were stimulated for either (i) apoptosis (TS), necroptosis (TSZ) or (ii) left untreated (Con). After four hours cells were treated with pMLKL (NSA) inhibitor or left untreated. **(C)** Cell viability was measured at different time point post cell death stimulation using A5/PI staining and analyzed by flow cytometry (mean ± sd). **(D)** Example of the flow cytometry smooth density plots are shown. Data are representative of one experiment from at least three independent experiments.
(TIFF)

S7 Fig (relevance to Fig 6). **(A)** U937 cells were first stained with CFSE prior to stimulation for apoptosis and necroptosis using a combination of TNFa, birinapant (SMAC mimetic) and zVAD. PS exposure was tested every 30 minutes until exposure reached 40% in both the apoptotic and necroptotic samples (determined by A5/PI staining). Cells were washed twice and re-suspended in DMEM before adding on IFN-g treated BMDMs at a 2.5:1 ratio. Phagocytosis was analyzed by flow cytometry and area under the curve was generated to compare kinetics. BMDMs with or without addition of live cells served as negative controls. Data are taken from three independent experiments. **(B-D)** U937 cells were treated as above. Cells were washed twice and re-suspended in DMEM before adding on TG peritoneal macrophages at a 2.5:1 ratio and **(B)** phagocytosis was analyzed by flow cytometry (mean ± sd). **(C)** Area under the curve was generated to compare kinetics. **(D)** Viability of the U937 cells, which were used as target to (B), is shown. TG peritoneal macrophages with no addition of cells or with addition of live cells served as negative controls. **(E-J)** Supernatants from the phagocytic TG peritoneal macrophages from (B) were collected and then analyzed for cytokines and chemokines using ELISAs. Statistic comparisons between each injected target cells were carried using ANOVA, followed by a Tukey’s multiple comparison test, * *P*≤*0.05* **P<*0.01*. **(K-M)** CFSE or Hoescht stained L929 cells were left untreated or stimulated for necroptosis (TSZ). PS exposure was tested every 30 minutes until reaching 40% in necroptotic samples (determined by A5/PI staining). A 1:1 ratio mix of live and necroptotic cells was i.p. injected (total of 2x10^6^ cells in 100ml per mouse). One hour post injection, F4/80 peritoneal cells were analyzed for phagocytosis of the live and/or necroptotic cells by flow cytometry. **(K)** Flow cytometry smooth density plots and gate strategy are shown. **(L)** Analysis of phagocytosis by staining of target cells (N = 2, mean ± SEM). **(M)** Summarize of phagocytosis by target cell phenotypes (N = 4, mean ± SEM).
(TIFF)

S1 Data (XLSX)

S2 Data (XLSX)

S3 Data (XLSX)

S4 Data (XLSX)

S5 Data (XLSX)

S6 Data (XLSX)

S1 Movie Kinetic of annexin V single positive cells in untreated U937 cells. U937 were treated as Fig 1D. Annexin V-FITC and PI were added to the cells and pictures were taken every 15 minutes using real time fluorescent microscopy (EVOS Fl Auto, Thermo Fisher Scientific).
(MP4)

S2 Movie Kinetic of annexin V single positive cells in U937-TS treated cells. U937 were treated as Fig 1D. Annexin V-FITC and PI were added to the cells and pictures were taken every 15 minutes using real time fluorescent microscopy (EVOS Fl Auto, Thermo Fisher Scientific).
(MP4)

S3 Movie Kinetic of annexin V single positive cells in U937-TSZ treated cells. U937 were treated as Fig 1D. Annexin V-FITC and PI were added to the cells and pictures were taken every 15 minutes using real time fluorescent microscopy (EVOS Fl Auto, Thermo Fisher Scientific).
(MP4)

S4 Movie Kinetic of annexin V single positive cells in U937-TSZ-nec1s treated cells. U937 were treated as Fig 1D. Annexin V-FITC and PI were added to the cells and pictures were taken every 15 minutes using real time fluorescent microscopy (EVOS Fl Auto, Thermo Fisher Scientific).
(MP4)

S5 Movie Kinetic of annexin V single positive cells in untreated HaCaT cells. HaCat cells were treated as Fig 1G. Annexin V-FITC and PI were added to the cells and pictures were taken every 30–45 min using real time fluorescent microscopy (IncuCyteZOOM).
(MP4)

S6 Movie Kinetic of annexin V single positive cells in HaCaT-TS treated cells. HaCat cells were treated as Fig 1G. Annexin V-FITC and PI were added to the cells and pictures were taken every 30–45 min using real time fluorescent microscopy (IncuCyteZOOM).
(MP4)

S7 Movie Kinetic of annexin V single positive cells in HaCaT-TSZ treated cells. HaCat cells were treated as Fig 1G. Annexin V-FITC and PI were added to the cells and pictures were taken every 30–45 min using real time fluorescent microscopy (IncuCyteZOOM).
(MP4)

S8 Movie Kinetic of annexin V single positive cells in HaCaT-TSZ-nec1s treated cells. HaCat cells were treated as Fig 1G. Annexin V-FITC and PI were added to the cells and pictures were taken every 30–45 min using real time fluorescent microscopy (IncuCyteZOOM).
(MP4)

S9 Movie Kinetic of annexin V single positive cells in HaCaT-TSZ-gsk872 treated cells. HaCat cells were treated as Fig 1G. Annexin V-FITC and PI were added to the cells and pictures were taken every 30–45 min using real time fluorescent microscopy (IncuCyteZOOM).
(MP4)

S10 Movie Kinetic of annexin V single positive cells in HaCaT-TSZ-NSA treated cells. HaCat cells were treated as Fig 1G. Annexin V-FITC and PI were added to the cells and pictures were taken every 30–45 min using real time fluorescent microscopy (IncuCyteZOOM).
(MP4)

## Abbreviations

A5: annexin V
APC: allophycocyanin
BMDM: bone marrow-derived macrophages
CC3: cleaved caspase-3
CFSE: carboxyfluorescein succinimidyl ester
DAMP: danger-associated molecular pattern
ECV: extracellular vesicle
IAP: inhibitor of apoptosis
LD: LiveDead
MCP1: monocyte chemoattractant protein-1
MFI: geometric mean fluorescence intensity
MLKL: mixed lineage kinase domain-like
Nec-1s: necroptotic inhibitor of RIPK1
NSA: necrosulfonamide
PI: propidium idodide
pMLKL: phosphorylated mixed lineage kinase-like
PS: phosphatidylserine
RIPK1: receptor-interacting protein kinase-1
RIPK3: receptor-interacting protein kinase-3
SD: standard deviation
SEM: scanning electron microscopy
SMAC: second mitochondrial-derived activator of caspase
TBZ: TNFα + birinapant + zVAD
TEM: transmission electron microscopy
TG: thioglycolate
TLR: toll-like receptor
TS: TNFα + SMAC mimetic
TSZ: TNFα + SMAC mimetic + zVAD
Z: Zombie
zVAD: Z-VAD-FMK
