# Supplementary figures and images for: Phosphatidylserine externalization, “necroptotic bodies” release, and phagocytosis during necroptosis
Source: PLoS Biol. 2017 Jun 26;15(6):e2002711. doi: 10.1371/journal.pbio.2002711 (PMC5501695; doi:10.1371/journal.pbio.2002711)

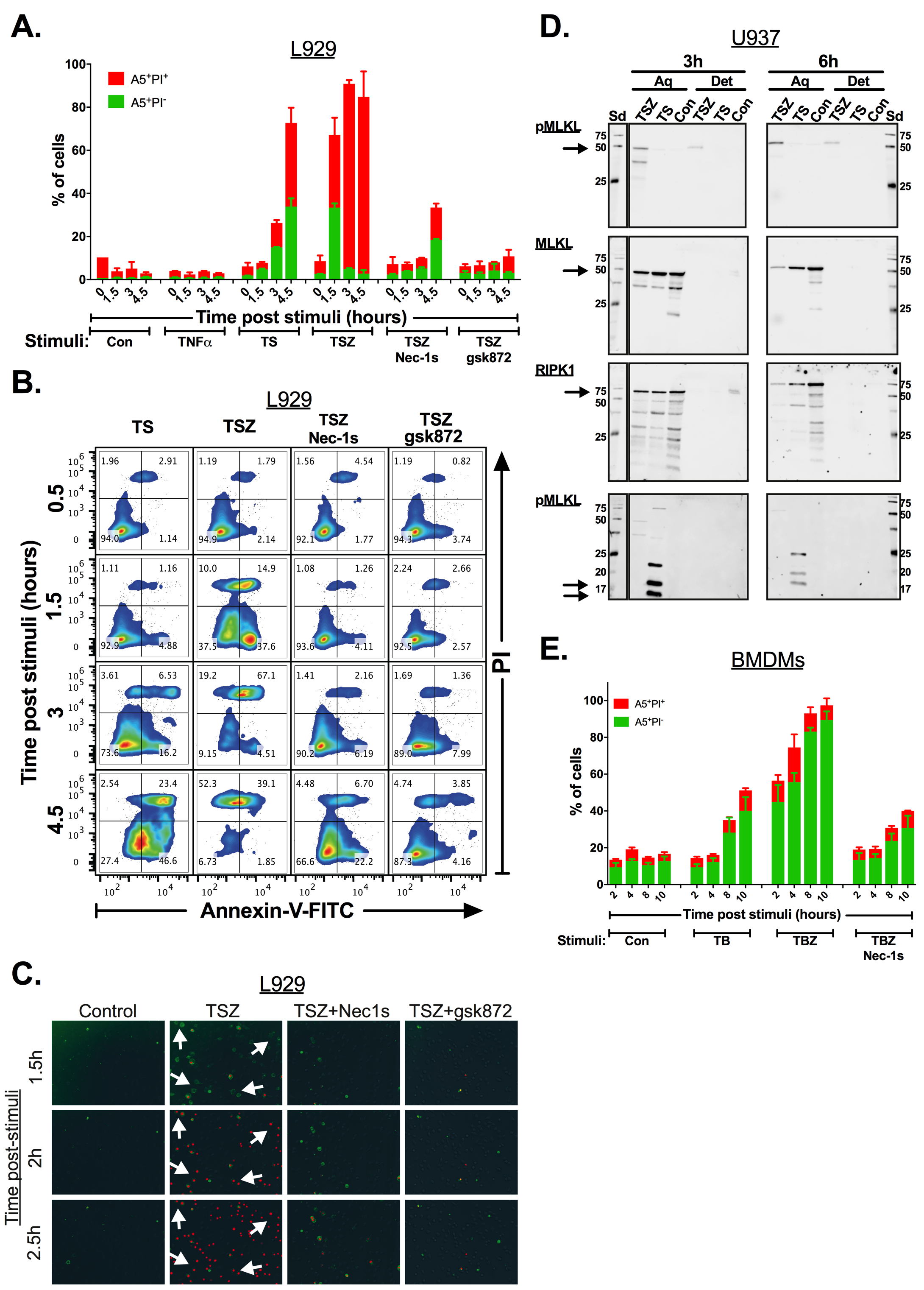

Supplement: S1 Fig — (A-C) L929 cells were stimulated using TNFa (T), SMAC mimetic (S) and zVAD (Z) as indicated or left unstimulated (Con). Where indicate RIPK1 (nec1s) and RIPK3 (gsk872) inhibitors were added to the cells 30 minutes prior to TSZ stimulation. (A) Cell viability was measured at different time point post cell death stimulation using annexin V/PI staining and analyzed by flow cytometry (mean ± sd). (B) Example of the flow cytometry smooth density plots are shown. (C) Example of the single A5-FITC-positive necroptotic cells using live microscopy are shown. (D) Three and six h post stimulation 106 U937 cells were harvested and two fractions were produced from every sample: Aqua (Aq)–hydrophilic fraction and Detergent (Det)—hydrophobic fraction. The kinetics of cell death key factors pMLKL, MLKL, RIPK1 and cleaved caspase 3 (CC3) were detected using western-blot. Sd–protein ladder. (E) BMDMs were stimulated for apoptosis (TB) and necroptosis (TBZ) or left unstimulated (Con). Where indicate RIPK1 (nec1s) and inhibitor was added to the cells 30 minutes prior to TBZ stimulation. (TIFF) [file pbio.2002711.s001.tiff]

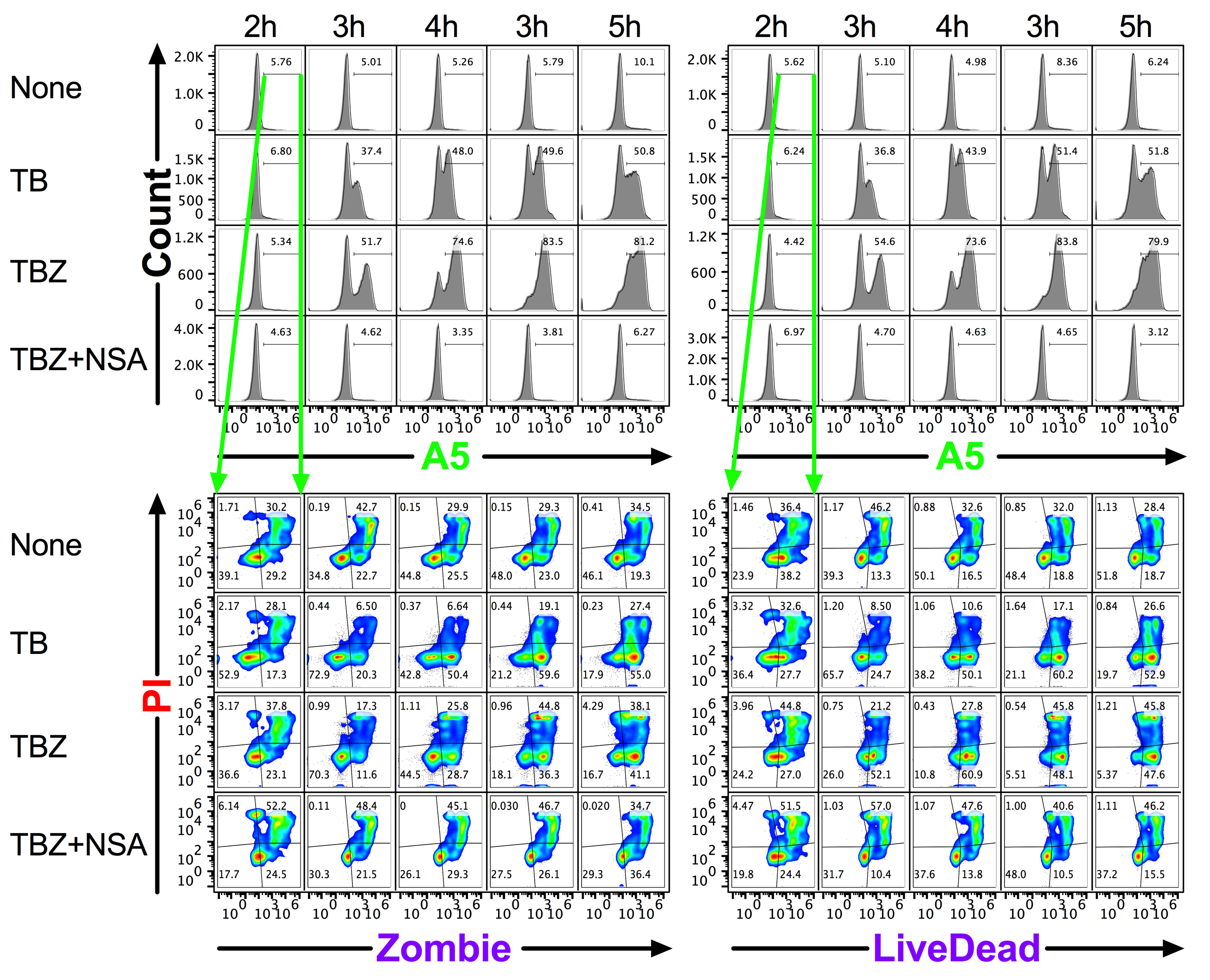

Supplement: S2 Fig — U937 cells were stimulated for either (i) apoptosis (TB), necroptosis (TBZ) or (ii) left untreated (Con). MLKL (NSA) inhibitor was added to the cells 30 minutes prior to TBZ stimulation. Illustration of flow cytometry gating strategy for A5, Zombie and PI (left panels) and A5, LiveDead and PI (right panels) triple staining. First, single cells were analyzed for A5 positivity (top histograms). A5 positive cells (green arrows) were further analyzed for Zombie and PI (lower left smooth density plots) and LiveDead and PI (lower right smooth density plots). (TIFF) [file pbio.2002711.s002.tiff]

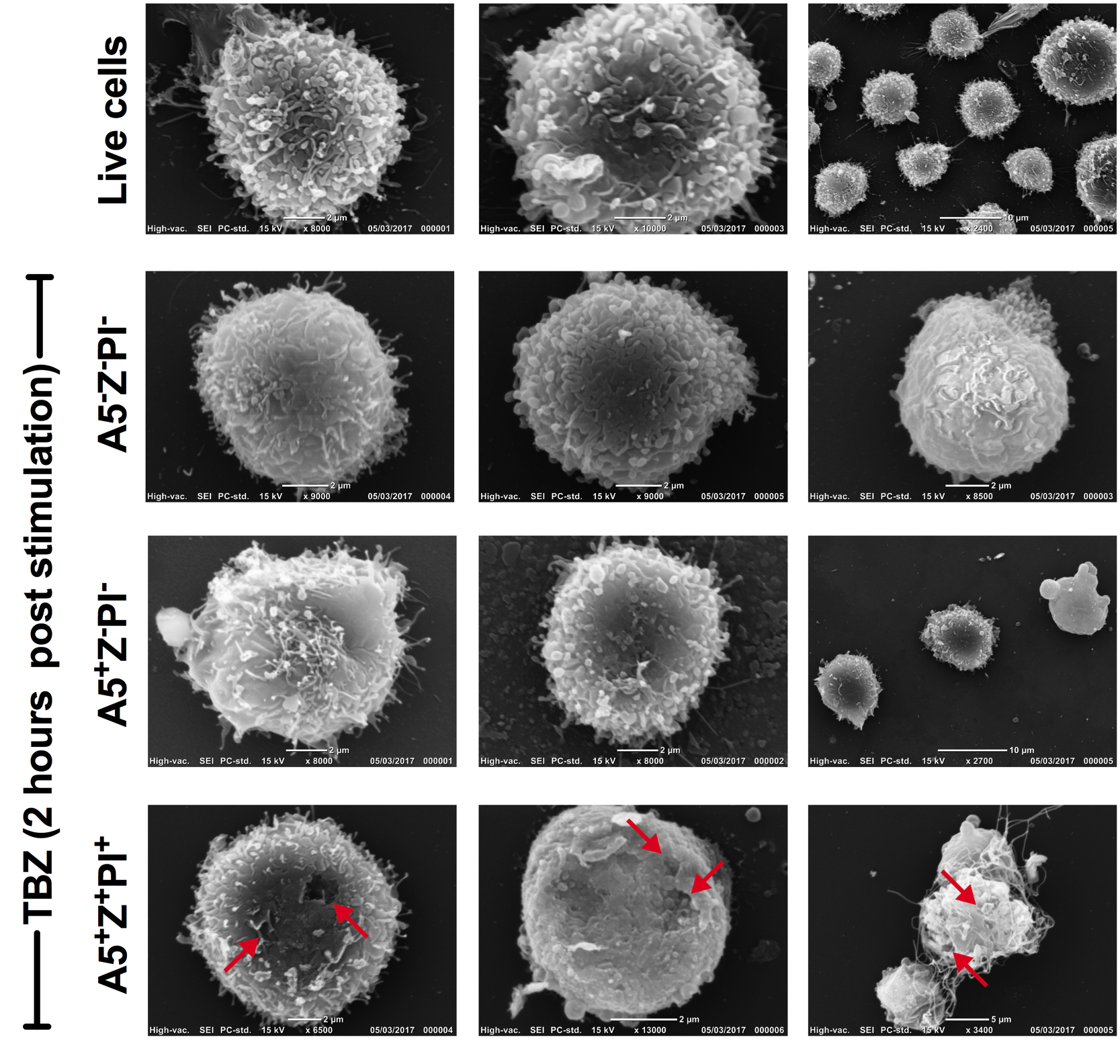

Supplement: S3 Fig — Necroptosis (TBZ) U937 cells were isolated into three different population according to their A5, Zombie and PI triple staining by FACSAria (BD Biosciences). Sorted cells and untreated cells (live cells) were fixed and prepared for SEM analysis. (TIFF) [file pbio.2002711.s003.tiff]

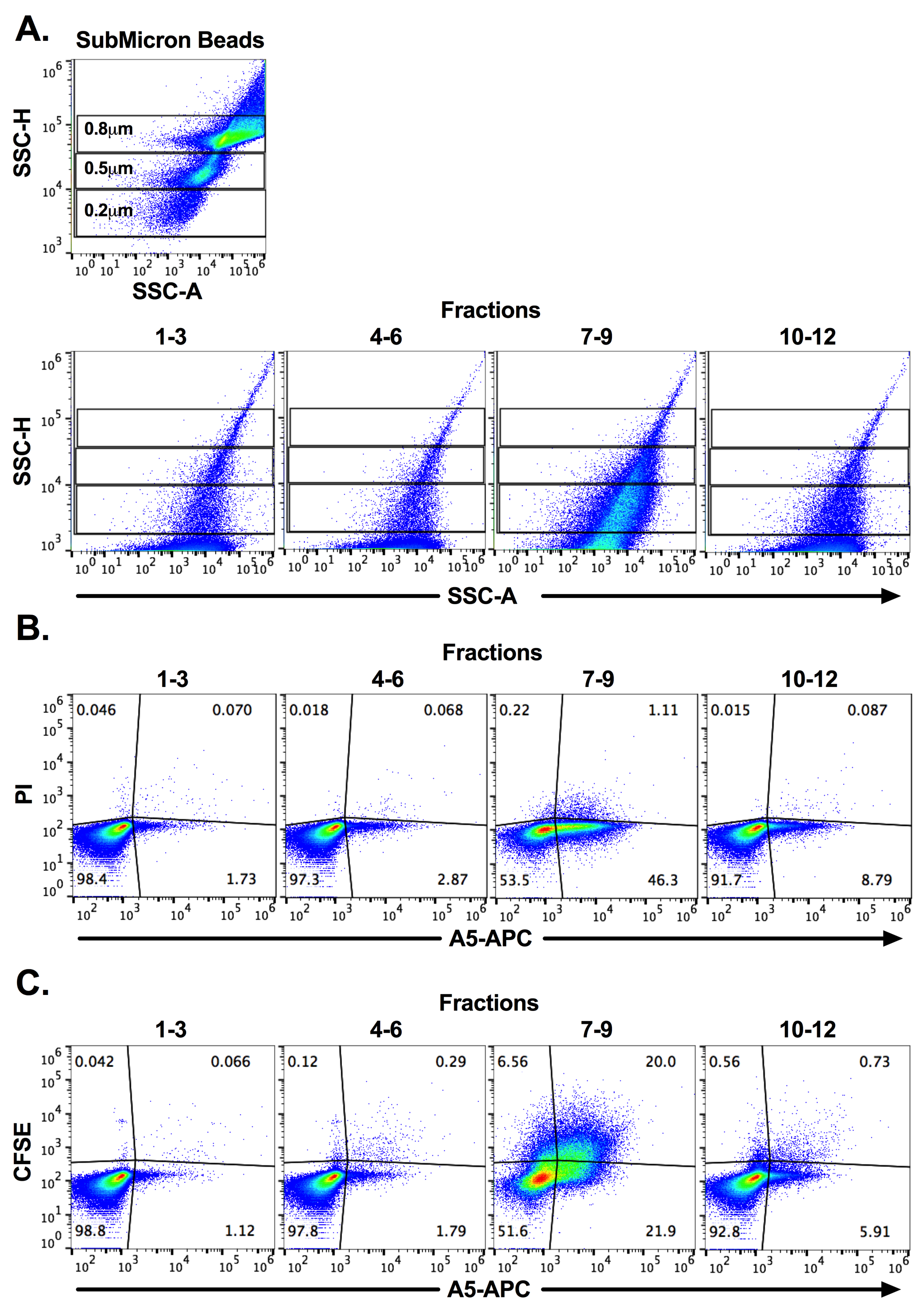

Supplement: S4 Fig — Extracellular vesicles (ECVs) from supernatants from CFSE labeled U937 necroptotic cells were isolated using size exclusion column (qEV, ZION). (A) The different fractions particles size was compared to known submicron beads. (B-C) The different fractions particles were further stained for A5 and PI and analyzed for A5, PI and CFSE using flow cytometry. (TIFF) [file pbio.2002711.s004.tiff]

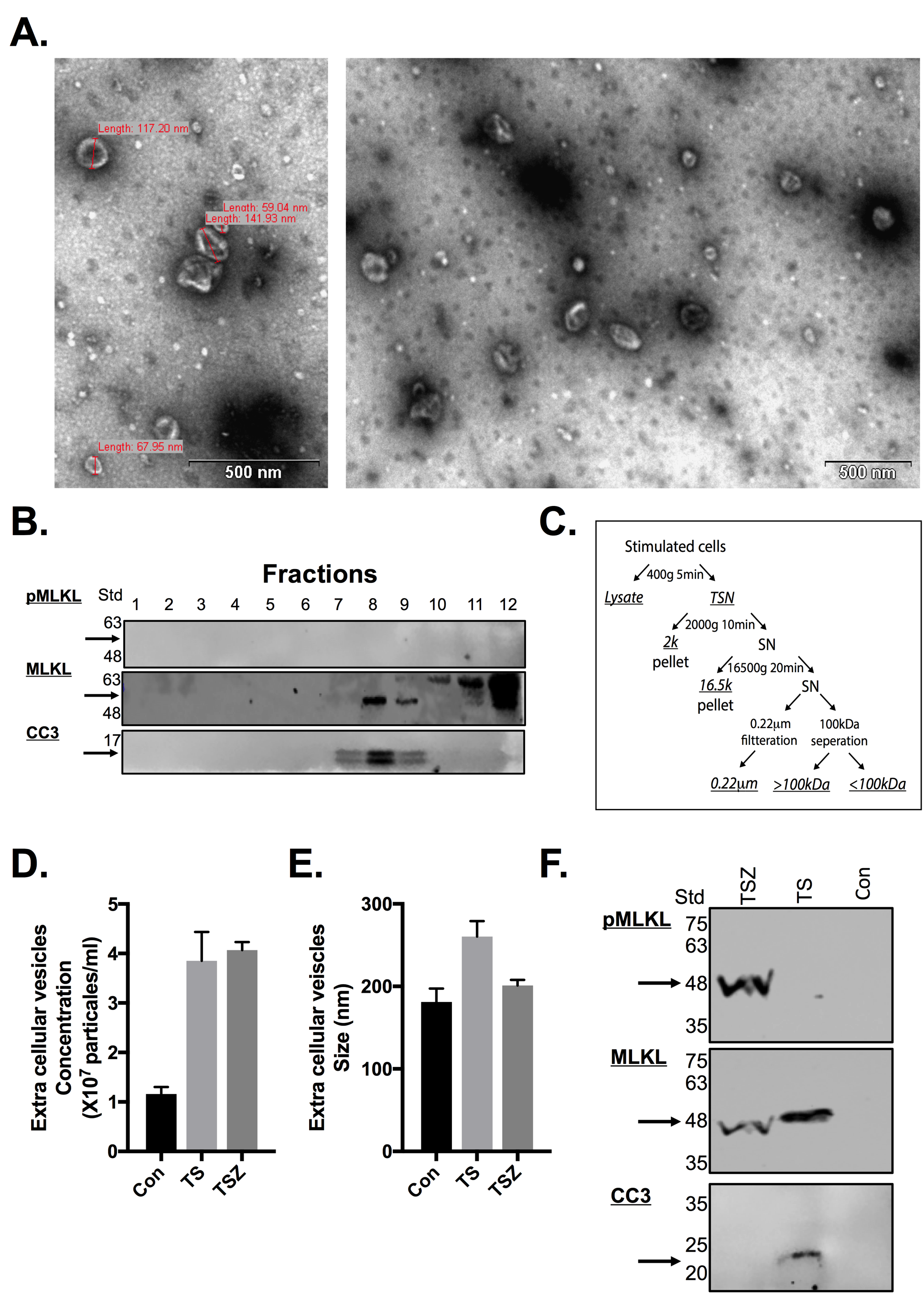

Supplement: S5 Fig — (A) Extra cellular vesicles (ECV) were isolated from necroptotic (TBZ) U937 cells by qEV Size Exclusion Column (IZon science). ECVs were prepared for transmission electron microscope (TEM) and images were captured on the JEM 1400plus transmission electron microscope (Jeol, Japan). (B) Supernatants from U937 apoptotic cells was fractionated using size exclusion column (qEV, ZION) and the cell death key factors pMLKL and cleaved caspase 3 (CC3) were detected using western-blot (SN–supernatants, Std–protein ladder). (C) Illustration of the fractionation of U937 treated cells and supernatants from Fig 4H. TSN-Total supernatant; SN- supernatant. (D-F) 5x10^6^ U937 cells were stimulated for either (i) apoptosis (TB), necroptosis (TBZ) or (ii) left untreated (None). ECVs from treated supernatants were isolated using ExoQuick kit (SBI, USA) and their concentration (D) and size (E) was analyzed using NanoSight. (F) Detection of pMLKL in the ECVs is shown. (TIFF) [file pbio.2002711.s005.tiff]

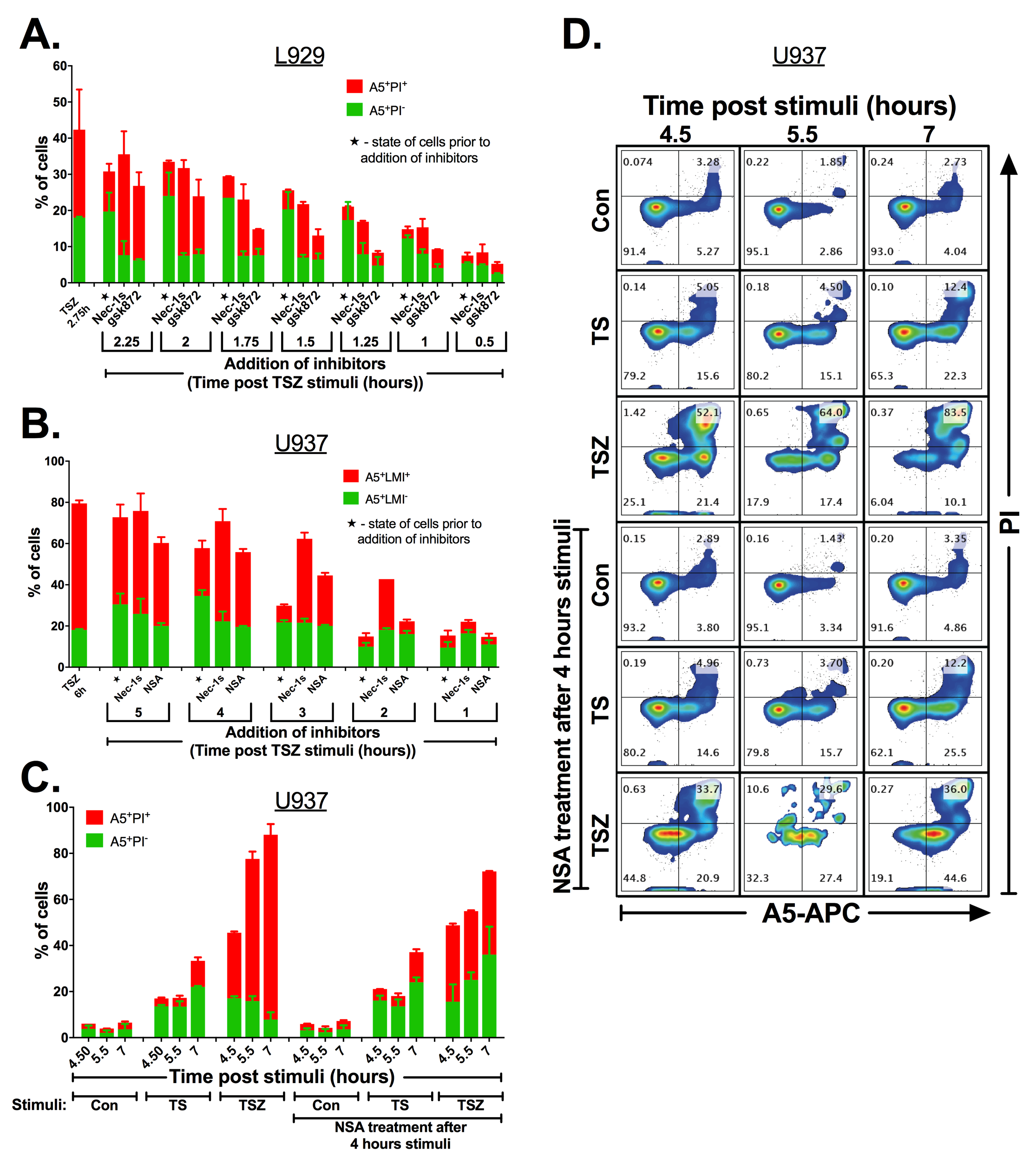

Supplement: S6 Fig — (A) L929 cells were stimulated for necroptosis (TSZ). From 30 minutes post stimulation, every 15 minutes cell viability was measured using A5/PI staining (indicate by «) prior to addition of RIPK1 (nec1s) or RIPK3 (gsk872) inhibitors. 2.75 hours post necroptosis induction cell viability was measured in all treatment using A5/PI staining and analyzed by flow cytometry. (B) U937 cells were stimulated for necroptosis (TSZ). Every hour post necroptosis stimulation cell viability was measured as below (indicate by «) prior to addition of RIPK1 (nec1s) or pMLKL (NSA) inhibitors. Six hours post necroptosis induction cell viability was measured in all treatment using A5/PI staining or A5/LiveDead (indicate as LMI positive) and analyzed by flow cytometry. (C-D) U937 cells were stimulated for either (i) apoptosis (TS), necroptosis (TSZ) or (ii) left untreated (Con). After four hours cells were treated with pMLKL (NSA) inhibitor or left untreated. (C) Cell viability was measured at different time point post cell death stimulation using A5/PI staining and analyzed by flow cytometry (mean ± sd). (D) Example of the flow cytometry smooth density plots are shown. Data are representative of one experiment from at least three independent experiments. (TIFF) [file pbio.2002711.s006.tiff]

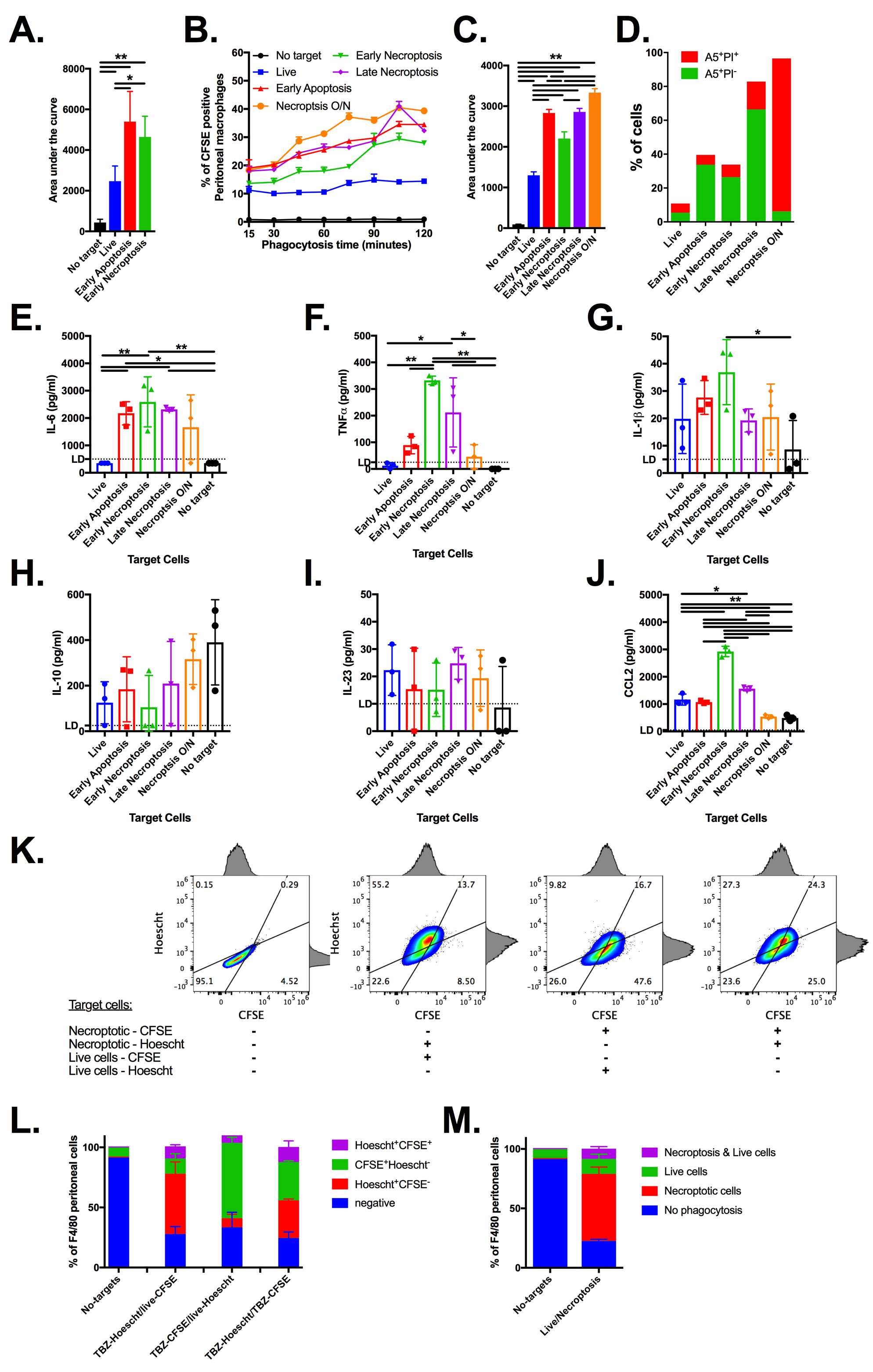

Supplement: S7 Fig — (A) U937 cells were first stained with CFSE prior to stimulation for apoptosis and necroptosis using a combination of TNFa, birinapant (SMAC mimetic) and zVAD. PS exposure was tested every 30 minutes until exposure reached 40% in both the apoptotic and necroptotic samples (determined by A5/PI staining). Cells were washed twice and re-suspended in DMEM before adding on IFN-g treated BMDMs at a 2.5:1 ratio. Phagocytosis was analyzed by flow cytometry and area under the curve was generated to compare kinetics. BMDMs with or without addition of live cells served as negative controls. Data are taken from three independent experiments. (B-D) U937 cells were treated as above. Cells were washed twice and re-suspended in DMEM before adding on TG peritoneal macrophages at a 2.5:1 ratio and (B) phagocytosis was analyzed by flow cytometry (mean ± sd). (C) Area under the curve was generated to compare kinetics. (D) Viability of the U937 cells, which were used as target to (B), is shown. TG peritoneal macrophages with no addition of cells or with addition of live cells served as negative controls. (E-J) Supernatants from the phagocytic TG peritoneal macrophages from (B) were collected and then analyzed for cytokines and chemokines using ELISAs. Statistic comparisons between each injected target cells were carried using ANOVA, followed by a Tukey’s multiple comparison test, * P≤0.05 **P<0.01. (K-M) CFSE or Hoescht stained L929 cells were left untreated or stimulated for necroptosis (TSZ). PS exposure was tested every 30 minutes until reaching 40% in necroptotic samples (determined by A5/PI staining). A 1:1 ratio mix of live and necroptotic cells was i.p. injected (total of 2x10^6^ cells in 100ml per mouse). One hour post injection, F4/80 peritoneal cells were analyzed for phagocytosis of the live and/or necroptotic cells by flow cytometry. (K) Flow cytometry smooth density plots and gate strategy are shown. (L) Analysis of phagocytosis by staining of target cells (N = 2, mean ± SEM) [file pbio.2002711.s007.tiff]
